# Suppression of amyloid-β fibril growth by drug-engineered polymorph transformation

**DOI:** 10.1016/j.jbc.2022.102662

**Published:** 2022-11-02

**Authors:** Sima Mafimoghaddam, Yuechuan Xu, Michael B. Sherman, Elena V. Orlova, Prashant Karki, Mehmet A. Orman, Peter G. Vekilov

**Affiliations:** 1 William A. Brookshire Department of Chemical & Biomolecular Engineering, University of Houston, Houston, Texas, USA; 2 Department of Biochemistry and Molecular Biology, University of Texas Medical Branch, Galveston, Texas, USA; 3 Department of Biological Sciences, Institute for Structural and Molecular Biology, Birkbeck University of London, London, UK; 4 Department of Chemistry, University of Houston, Houston, Texas, USA

**Keywords:** Alzheimer's disease, amyloid fibril growth, suppression of fibrillization, molecular mechanism of fibrillization, neurotoxicity, Aβ, amyloid β, Aβ40, 40-residue Aβ isoform, AD, Alzheimer’s disease, AFM, atomic force microscopy, MTT, 3-(4,5-dimethylthiazol-2-yl)-2,5-diphenyltetrazolium bromide, ThT, thioflavin T

## Abstract

Fibrillization of the protein amyloid β is assumed to trigger Alzheimer’s pathology. Approaches that target amyloid plaques, however, have garnered limited clinical success, and their failures may relate to the scarce understanding of the impact of potential drugs on the intertwined stages of fibrillization. Here, we demonstrate that bexarotene, a T-cell lymphoma medication with known antiamyloid activity both *in vitro* and *in vivo*, suppresses amyloid fibrillization by promoting an alternative fibril structure. We employ time-resolved *in situ* atomic force microscopy to quantify the kinetics of growth of individual fibrils and supplement it with structure characterization by cryo-EM. We show that fibrils with structure engineered by the drug nucleate and grow substantially slower than “normal” fibrils; remarkably, growth remains stunted even in drug-free solutions. We find that the suppression of fibril growth by bexarotene is not because of the drug binding to the fibril tips or to the peptides in the solution. Kinetic analyses attribute the slow growth of drug-enforced fibril polymorph to the distinctive dynamics of peptide chain association to their tips. As an additional benefit, the bexarotene fibrils kill primary rat hippocampal neurons less efficiently than normal fibrils. In conclusion, the suggested drug-driven polymorph transformation presents a mode of action to irreversibly suppress toxic aggregates not only in Alzheimer’s but also potentially in myriad diverse pathologies that originate with protein condensation.

The accumulation of fibrils and plaques of the protein amyloid β (Aβ) in patients’ brains is deemed a trigger of Alzheimer’s disease (AD) and the related cerebral amyloid angiopathy (1, 2, 3). A recent clinical study found a correlation between the brain loci of entangled tau, activated microglia, and Aβ accumulation and concluded that accumulated Aβ may potentiate the activation of microglia and then synergistically cooperate with the activated microglia to promote the propagation of tau tangles and increase the severity of cognitive symptoms (4). In futher support of the agency of amyliud fibrils and palques, a substantial fraction of potential AD drugs currently in phase 3 clinical trials aims at Aβ fibrils and plaques (5, 6, 7, 8, 9, 10, 11, 12, 13, 14, 15, 16, 17). Notably, select approaches to delay or reverse fibrillization in the clinic (18, 19, 20, 21) have accumulated limited success (22, 23, 24, 25), which has been construed as a suggestion that alternative pathophysiological pathways may be preferred as intervention targets (23, 26, 27, 28).

The limited success of some of the to date the clinical approaches based on suppression of Aβ fibrils and plaques may be partially attributed to the insufficient insight into how potential drugs interfere in the complex network of intertwined processes that generate amyloid aggregates (27, 29, 30). In the first step of fibrillization, several peptide monomers jointly search for a stable structure (31). This step is identified as nucleation, and it spawns a population of small aggregates that may already be neurotoxic (1). The assembled nuclei grow into linear fibrils by adding peptide chains from the solution (32, 33). As the fibrils prolongate, they may branch by secondary nucleation or fracture to release peptide oligomers, which, in turn, boost nucleation (27, 34). This complex network of events can generate vast amounts of fibrils and oligomers that spread disease throughout patients’ brains. Drugs, such as bexarotene, a cutaneous T-cell lymphoma medication (35) with antiamyloid activity both *in vitro* and *in vivo* (30, 36), may intervene at any of the constituent processes. They may bind to peptide monomers (Fig. 1*A*) or oligomers (Fig. 1*B*) (30) and delay or fully stunt further aggregation. They may cap the tips (Fig. 1*C*) or affix to the sides of growing fibrils to inhibit growth and delay branching and fragmentation.

**Figure 1:**
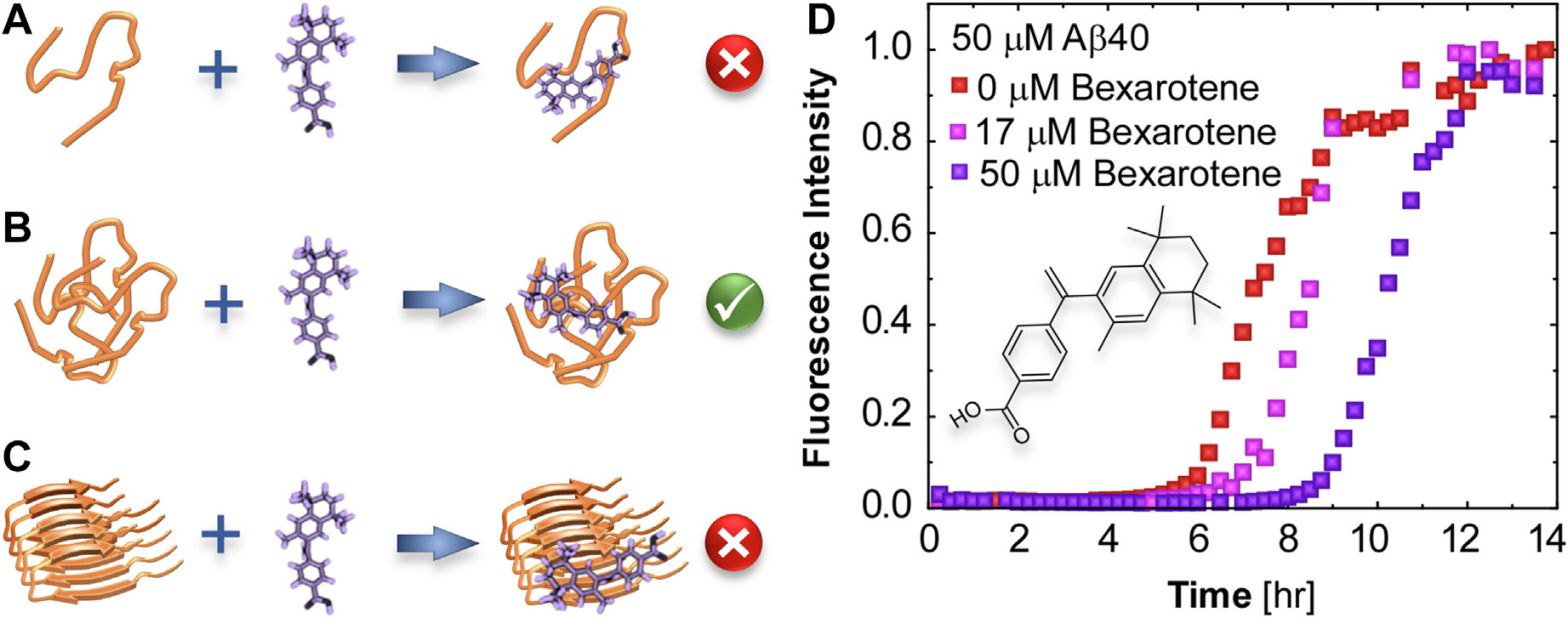
**Bexarotene effects on Aβ40 fibrillization kinetics.***A*–*C*, schematic illustrations of binding of bexarotene (*purple*) to an Aβ40 monomer (*gold*) (*A*) and an Aβ40 oligomer (*B*) and capping of the fibril tip (*C*). *D*, the evolution of thioflavin T (ThT) fluorescence at 488 nm in 50 μM solutions of Aβ40 in the absence and presence of bexarotene at 17 and 50 μM. *Inset*, the chemical structure of bexarotene. Aβ, amyloid β; Aβ40, 40-residue Aβ isoform.

To unveil the molecular mechanism employed by a drug to interfere in amyloid aggregation, we focus on the 40-residue Aβ isoform (Aβ40). Aβ40 dominates the amyloid plaques in the meninges of AD patients (37, 38) and has been found in plaques extracted from patients with both presymptomatic and symptomatic AD (3, 39). Aβ40 also accumulates in the brain vasculature, where it causes cerebral amyloid angiopathy (38). We focus on the growth rates of individual fibrils, which we quantify by time-resolved *in situ* atomic force microscopy (AFM). The kinetic data afford the opportunity to identify the steps of the molecular mechanism of peptide incorporation at the fibril tips and evaluate the governing thermodynamic and kinetic parameters (40, 41). In contrast to previous AFM work, we base our conclusions on statistics over multiple fibrils and emphasize reproducible behaviors. We extend the kinetics measurements to fibril dissolution in peptide-free solutions. Finding the lowest concentration for net fibril growth defines the fibril solubility and quantifies the equilibrium constant for fibril growth and the related standard free energy for fibrillization. We supplement the kinetic insights with fibril structure characterization by cryo-EM. To elucidate how the findings of the chemical mechanism of Aβ fibrillization may fit in the context of brain damage and disease, we measure the toxicity of fibrils formed in the presence of bexarotene to neurons.

## Results and discussion

### Passivity of bexarotene to the fibril tips

To monitor the evolution of fibrillization and the overall impact of bexarotene, we employed the fluorescence intensity emitted at 488 nm by the dye thioflavin T (ThT) in the presence of Aβ sheets, which is expected to scale the total amyloid mass (42). The initial period of negligible fluorescence from Aβ40 solutions with added ThT (Fig. 1*D*) manifests slow nucleation (43, 44). The addition of bexarotene further extends this period (Fig. 1*D*), announcing that bexarotene suppresses Aβ40 fibril nucleation (45, 46). Spectroscopic tests reveal (Fig. S2) that bexarotene does not perceptibly bind to Aβ40 monomers (Fig. 1*A*), analogously to its undetectable binding to the similar Aβ42 (47, 48); notably, the Aβ40 sequence replicates the first 40 amino acid residues of Aβ42, and both Aβ42 and Aβ40 are unstructured (3). With this, the observed nucleation delay by bexarotene implies that the drug interacts with the only other Aβ40 species present during nucleation, Aβ40 peptide oligomers (Fig. 1*B*), analogously to its association to Aβ42 oligomers (30).

**Figure 2:**
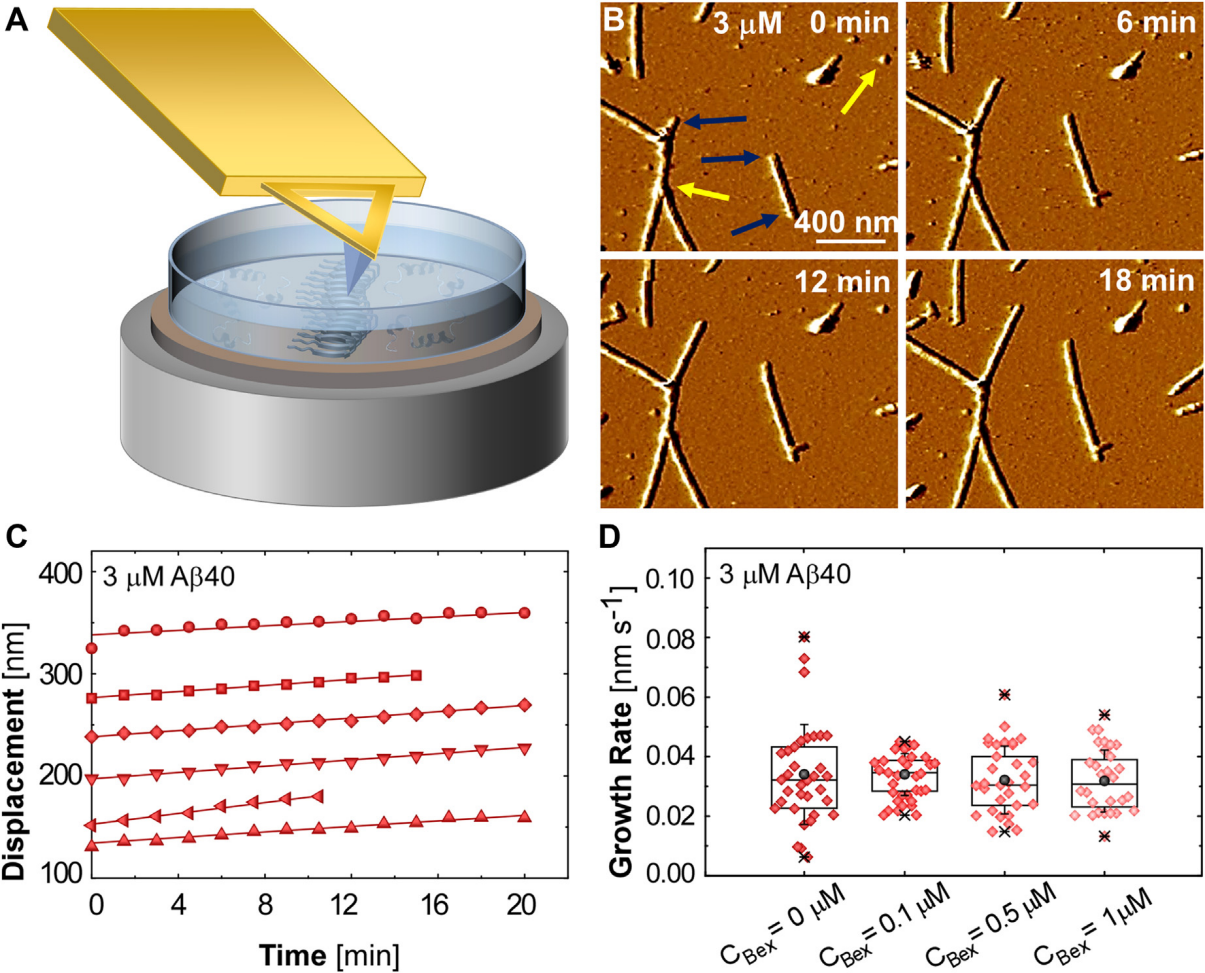
**Bexarotene effects on fibril growth.***A*, schematic of *in situ* AFM imaging of a fibril deposited on a substrate and submerged in an Aβ40 solution. *B*, AFM images of Aβ40 fibrils recorded *in situ* during growth in a 3 μM Aβ40 solution at indicated times after the first image. *Yellow arrows* point to immobile reference points; *navy arrows* indicate growing fibril tips. *C*, the displacements of six fibril tips, depicted with distinct symbols, growing in 3 μM solutions measured from sequences of AFM images as in *B*. The slopes of the best linear-regression fits define the growth rate of the monitored fibril. *D*, jitter plots of the rates of fibril growth in a 3 μM solution after adding bexarotene at indicated concentrations *C*_*Bex*_. About 30 measurements, similar to those illustrated in *C*, are shown for each *C*_*Bex*_. *Crosses* mark the slowest and fastest growth rates, *black spheres* indicate the average values, and *capped vertical bars* display the standard deviations of the datasets. *Lower*, *median*, and *upper horizontal lines* represent 25, 50, and 75% of the data points, respectively. ANOVA and Kruskal–Wallis tests (Tables S3 and S5) support the hypothesis that the average growth rates in the presence of 0.1, 0.5, and 1.0 μM bexarotene are equal to those measured in the absence of bexarotene. Aβ, amyloid β; Aβ40, 40-residue Aβ isoform; AFM, atomic force microscopy.

The upturn of fluorescence intensity (Fig. 1*D*) that follows the period dominated by nucleation reports contributions to fibril mass from concurrent fibril growth, branching, and fragmentation that are hard to deconvolute. To judge whether bexarotene inhibits the first of these steps, fibril growth, we turn to time-resolved *in situ* AFM (41) (Fig. 2, *A–D*). We deposit fibrils preformed in the absence of bexarotene on mica and monitor the growth of the fibril tips along the fibril axis with respect to immobile reference points (Fig. 2*B*) in Aβ40 solutions with known concentrations. We evaluate the fibril growth rate as the slope of the time correlation of the fibril tip displacement (Fig. 2*C*) (41). Our previous examination of this method (41) revealed that the measured fibril growth rates and solubilities were similar to those determined from fibrils suspended in solution (33). This agreement signifies that interactions with the substrate that may strain the fibrils or assist the supply of monomers to the fibril tips do not modify the growth rates (41). The addition of bexarotene at three concentrations up to 1 μM (which exceeds the concentration of fibril tips by many orders of magnitude) does not modify the average growth rates of fibrils nucleated in the absence of this drug (Fig. 2*D*). Fibril growth comprises association of peptide chains to fibril tips. The passivity of bexarotene to fibril growth concurs with the lack of bexarotene–Aβ40 peptide binding (Fig. S2) and also advocates that bexarotene interactions with the tips or alternative sites of the fibrils are weak (Fig. 1*C*).

### Bexarotene modifies the structure of the fibrils nucleated in its presence

**Figure 3:**
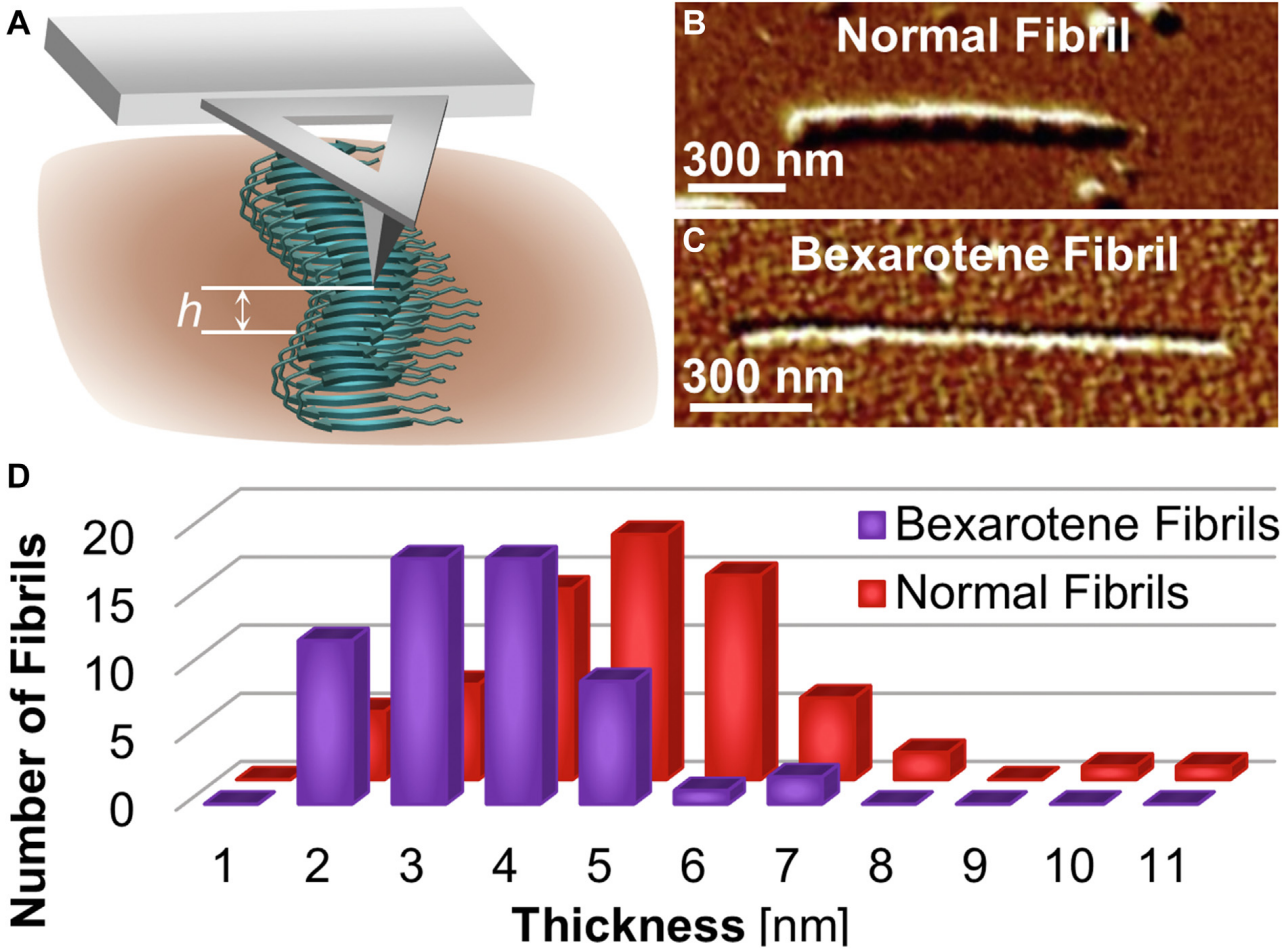
**AFM characterization of the morphology of normal and bexarotene fibrils.***A*, schematic of the AFM measurement of fibril thickness *h* as height deviation from the substrate. *B* and *C*, AFM images of normal (*B*) and bexarotene (*C*) fibrils. *D*, thickness distributions of normal (*red*, *back row*) and bexarotene (*purple*, *front*) fibrils growing at *C*_*Aβ40*_ = 3 μM obtained from, respectively, 68 and 60 thickness measurements by AFM. Aβ, amyloid β; Aβ40, 40-residue Aβ isoform; AFM, atomic force microscopy.

AFM and cryo-EM characterization of the morphology of fibrils nucleated in the presence and absence of bexarotene advance that bexarotene not only delays fibril nucleation (Fig. 1*D*) but also drives the assembly of a distinct Aβ40 fibril structure. Aβ40 assembles into fibrils with diverse structures (often called polymorphs), in which the peptide chains fold into unique conformations (32, 37, 49, 50, 51). The divergent molecular arrangements of the polymorphs dictate specific mesoscopic morphologies identifiable by AFM and cryo-EM. AFM measures the fibril thickness as the maximum separation of the AFM probe from the substrate, averaged from 10 scans perpendicular to the fibril axis (Fig. 3*A*). Statistics over 60 fibrils nucleated in the presence of bexarotene (Fig. 3*C*) and 68 fibrils nucleated without the drug (Fig. 3*B*) reveal that bexarotene fibrils—with average thickness 4 ± 1 nm—are, on the average, thinner than the normal fibrils, whose average thickness is 5 ± 1 nm (Fig. 3*D* and Table S1).

**Figure 4:**
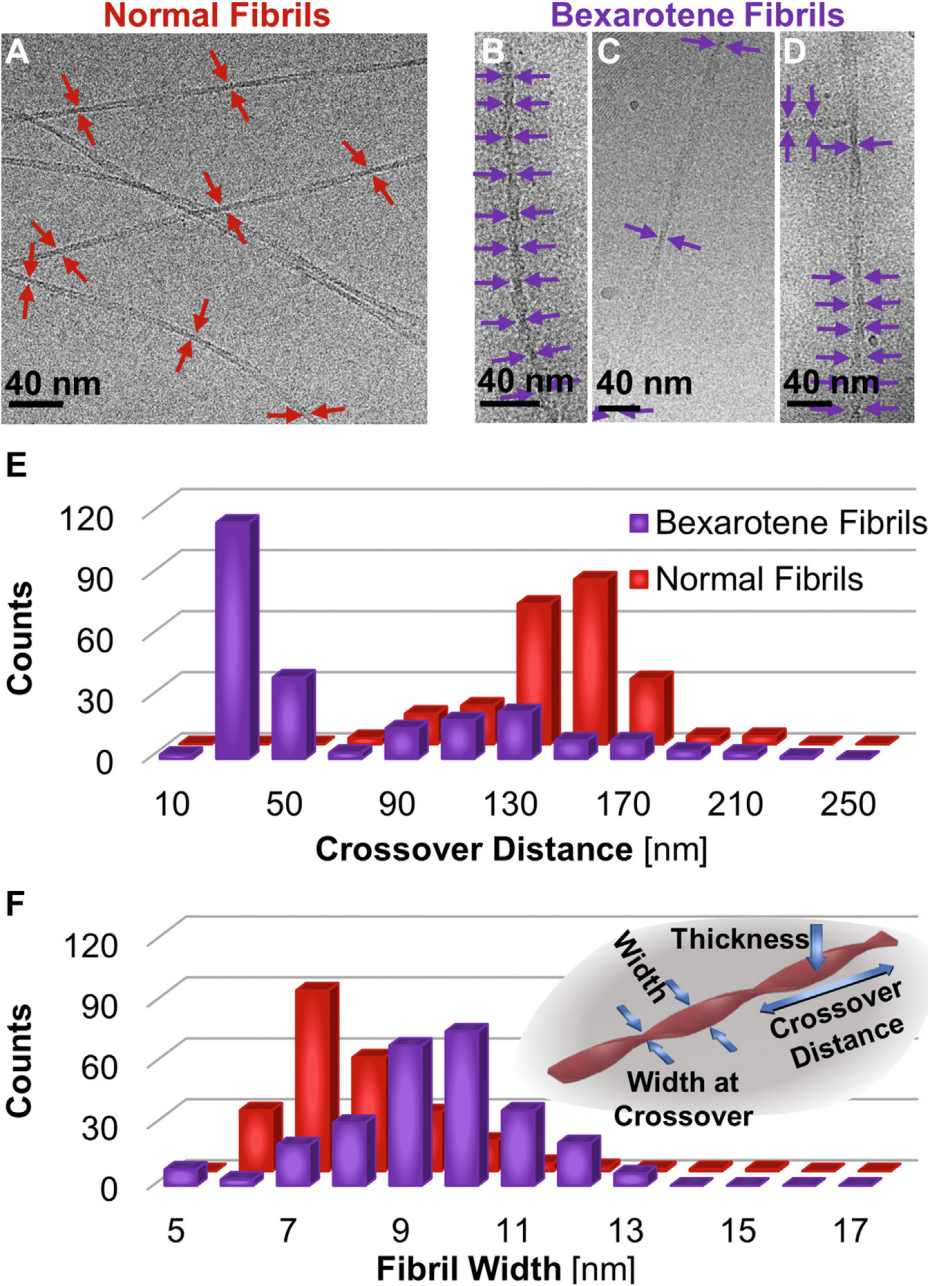
**Cryo-EM characterization of the morphology of normal and bexarotene fibrils.***A*–*D*, cryo-EM micrographs of normal (*A*) and bexarotene (*B*–*D*) fibrils. The crossover points are indicated by *arrows*. *E* and *F*, distributions of crossover distances (*E*) and fibril widths (*F*) for normal (*red*, *back row*) and bexarotene (*purple*, *front*) fibrils. *Inset*, a schematic of a twisted fibril with *arrows* delineating thickness, crossover distance, fibril width, and width at crossover point. ANOVA tests (Table S3) for the distributions in *E* and *F* support the hypotheses that the average thicknesses, in *E*, and fibril widths, in *F*, for normal fibrils are distinct from the respective characteristics of bexarotene fibrils.

Cryo-EM (Fig. 4, *A–F*) demonstrates that both types of fibrils appear as twisted flat strips and affords the opportunity to quantify two distinguishing characteristics of the fibril morphology: the crossover distance and the fibril width (Fig. 4*F*, *inset*). The cryo-EM micrographs (Fig. 4, *A–D*) establish that the examined normal fibrils structure rather uniformly, with an average crossover distance of ca. 140 nm and average fibril width of ca. 8 nm (Fig. 4, *A*, *E* and *F*). By contrast, the dominant subpopulation of the bexarotene fibrils exhibits a substantially shorter average crossover distance of ca. 30 nm (Fig. 4*E* and Table S1) and an exaggerated average width of ca. 10 nm (Fig. 4*F* and Table S1); a minority subpopulation presents morphological characteristics similar to those of normal fibrils, whose nucleation may have been unaffected by bexarotene. The distributions of the widths at the crossover points of both normal and bexarotene fibrils (Fig. S3) approximately match the respective distributions of AFM-determined thicknesses (Fig. 3*D*); the two measures roughly correspond to the same fibril dimension owing to the distinctive perspectives of the two methods (Fig. 4*F*, *inset*). Interestingly, lone bexarotene fibrils feature variable crossover length that might expose the merging of two independently nucleated fibrils (Fig. 4*D*).

### Distinct molecular mechanism of growth of fibrils with bexarotene-engineered structure

**Figure 5:**
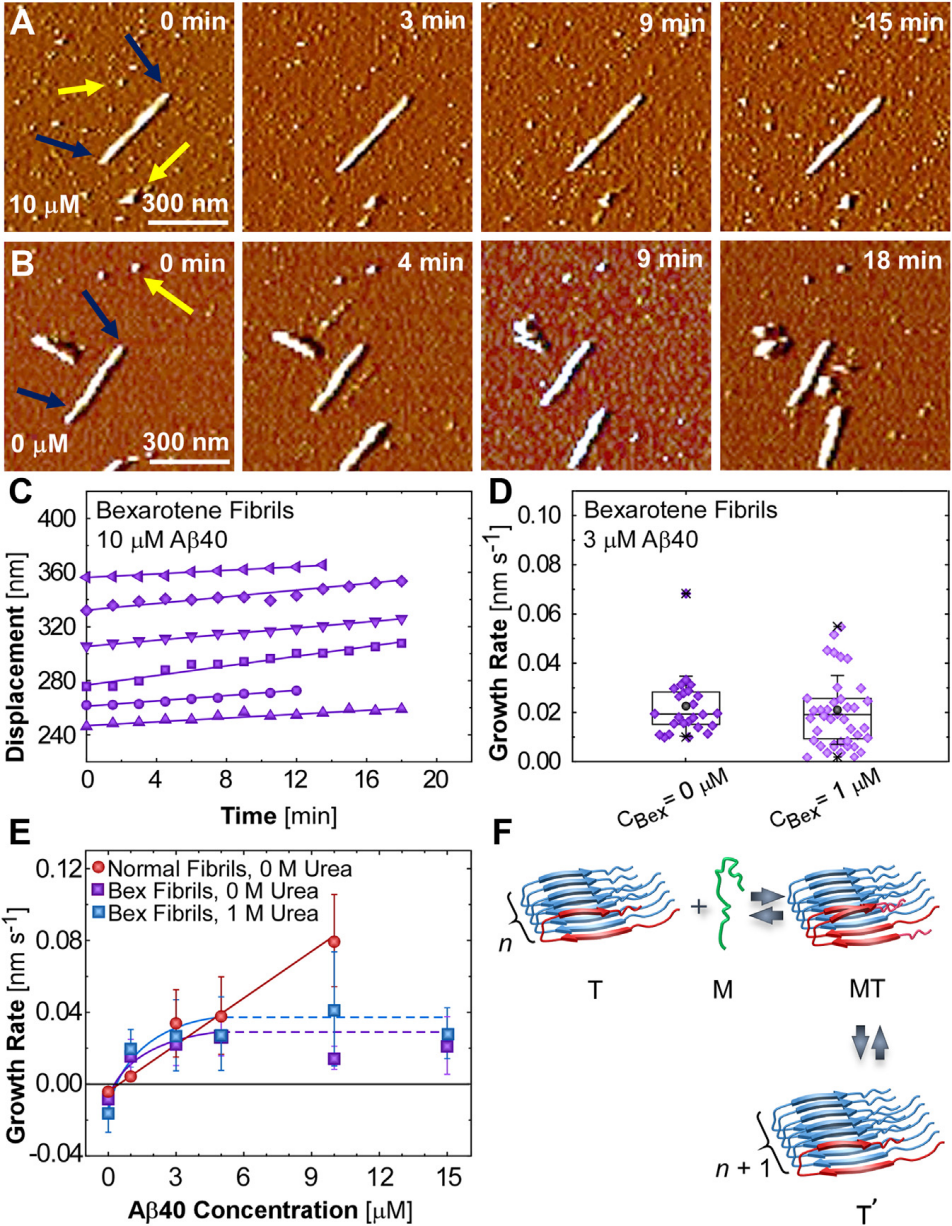
**The growth of bexarotene fibrils.***A* and *B*, *in situ* AFM images of bexarotene fibrils growing in a 10 μM Aβ40 solution (*A*) and dissolving in a peptide-free solution (*B*). *Yellow arrows* indicate immobile reference points, and *navy arrows* point to fibril tips that grow or dissolve. *C*, evolutions of the displacements of six bexarotene fibril tips at *C*_*Aβ40*_ = 10 μM. *D*, jitter plots of the growth rates of bexarotene fibrils at *C*_*Aβ40*_ = 3 μM in the absence and presence of 1 μM bexarotene. ANOVA tests (Table S3) support the hypothesis that the two average growth rates are equal. *E*, correlations between *C*_*Aβ40*_ and the growth rates of normal fibrils (*red*, data from Ref. (41)) and bexarotene fibrils in the presence of 1 M urea (*blue*) and in urea-free solutions (*purple*). Error bars indicate standard deviations from the averages of 20 to 30 measurements for each composition and fibril type. *Solid lines* depict best fits to a kinetic model; *horizontal dashed lines* are extensions for high *C*_*Aβ40*_. *F*, schematic illustration of the association of an Aβ40 monomer, M, *green*, to a complex at the fibril tip with a conformation distinct from the one in the fibril bulk, T, *red*, followed by the integration of one peptide chain into the fibril to produce a longer fibril T^’^. Aβ40 chains with native contacts in the fibril bulk are drawn in *blue*. Aβ, amyloid β; Aβ40, 40-residue Aβ isoform; AFM, atomic force microscopy.

The rate of fibril growth as solute peptides associate to the fibril tip and its correlations with the concentrations of the peptide and a denaturant present sensitive indicators of the molecular-level processes that comprise growth (40, 41). Fibrils with bexarotene-enforced structure exhibit unique kinetics of growth and dissolution, which combine features shared with normal fibrils (41) and dramatically divergent behaviors. Time-resolved *in situ* AFM images (Fig. 5*A*) reveal that bexarotene fibrils grow at steady rates (Fig. 5*C*) when exposed to solutions of Aβ40 without bexarotene. In further analogy to normal fibrils (41), when submerged in a buffer with no Aβ40, bexarotene fibrils steadily shorten (Figs. 5*B* and S4), reporting the release of peptide chains from the fibril tips into the solution. The line connecting the negative rate of fibril dissolution and the positive growth rate crosses the line of zero growth (Fig. 5*E*) at *C*_*e*_, the concentration at which the fibrils are in equilibrium with the solution, which is often called solubility (40, 41). The solubility of bexarotene fibrils, 0.33 ± 0.06 μM (Table S2), is somewhat lower than the solubility of normal fibrils, 0.44 ± 0.07 μM (40, 41). The growth rate of bexarotene fibrils at *C*_*Aβ*40_ = 1 μM is faster than the respective rate for normal fibrils (Fig. 5*E*). Importantly, adding bexarotene to the Aβ40 solution does not inhibit or promote the growth of fibrils with structure imposed by bexarotene (Fig. 5*D*). The preserved growth rate in the presence of bexarotene announces that the drug is indifferent to the tips or any other features of the bexarotene fibrils involved in growth—just as to normal fibrils (Fig. 2*D*).

The growth of bexarotene fibrils at *C*_*Aβ*40_ greater than 3 μM is insensitive to increasing *C*_*Aβ*40_ and substantially deviates from the growth of normal fibrils (Fig. 5*E*). We relate the saturating dependence of the growth rate on *C*_*Aβ*40_ to our previous finding that the incorporation of a solute peptide into the fibril tip occurs in two steps (40). First, an incoming peptide associates to a complex occupying the fibril tip and composed of one or more chains that have conformations distinct from those in the fibril bulk. In the second step, one of the peptides within that complex rearranges to the bulk fibril structure (Fig. 5*F*) (40). The similarities and differences of this scenario to a “lock-and-dock mechanism” put forth by simulations (52, 53, 54, 55) are discussed (40).

We assume that a similar two-step pathway guides peptide association to bexarotene fibril tips. In the absence of an atomic structure of the bexarotene fibrils, complete molecular modeling of the relevant dynamics that may detail the peptide association to a complex, in which the peptide chains assume conformation distinct from the one in the fibril bulk, and then its transformation to the native structure, as done for normal fibrils (40), would be challenging. Instead, we analyze the *R*(*C*_*Aβ*40_) correlations for bexarotene and normal fibrils to reveal that essential features of the two steps differ for the two fibril structures. Dimers and higher order oligomers, extant in Aβ solutions, reside in equilibrium with a majority of monomers (56, 57). Our previous results inform that normal Aβ40 fibrils grow by sequential addition of monomers (40, 41) and bolster the assumption that bexarotene fibrils also grow by monomer association. We model the two-step incorporation of Aβ40 monomers *M* into fibril tips *T* with a Michaelis–Menten-type sequence of two reactions, as in Ref. (33), $M+T\begin{array}{l} k_{1}\\ ⇌\\ k_{-1} \end{array}MT\begin{array}{l} k_{2}\\ ⇌\\ k_{-2} \end{array}T^{\prime }$, where *k*_*i*_ are the respective rate constants, *MT* denotes the intermediate complex at the tip, and *T'* are tips with an added peptide (Fig. 5*F*). Mass preservation relates the fibril average growth rate *R* to the rate of consumption of monomers $-\frac{d\left[M\right]}{dt}$, $R=-\frac{a}{C_{T}}\frac{d\left[M\right]}{dt}$ (see Supporting information), where *a* is the contribution of one peptide chain to the fibril length and *C*_*T*_ is the total concentration of fibril tips. We exploit that the solubility *C*_*e*_ relates to the equilibrium constants *K*, of the entire process, and *K*_*i*_, of the two constituent reactions, and the rate constants *k*_*i*_, $C_{e}≅{\left[M\right]}_{e}=K^{-1}=K_{1}^{-1}K_{2}^{-1}=\frac{k_{-1}k_{-2}}{k_{1}k_{2}}$ (the first equality reflects the dominance of monomers in the solution (56, 57), and the second is afforded by the reversibility of fibril growth and the equality $\left[T\right]=\left[T^{\prime }\right]$ (40)) and arrive (see Supporting information) at

$$R=a\left(\frac{k_{1}k_{2}\left(C_{A\beta 40}-C_{e}\right)}{k_{1}C_{A\beta 40}+k_{-1}+k_{2}+k_{-2}}\right)$$

At low *C*_*Aβ*40_ or if the reactions that involve the intermediate complex *MT* are fast, $k_{1}C_{A\beta 40}<k_{-1}+k_{2}+k_{-2}$. We retrieve a linear growth rate law $R=ak_{a}\left(C_{A\beta 40}-C_{e}\right)$, where $k_{a}=k_{1}k_{2}{\left(k_{-1}+k_{2}+k_{-2}\right)}^{-1}$, which describes the *R*(*C*_*Aβ*40_) correlation for normal fibrils (40, 41) (Fig. 5*E*). In cases where the intermediate complex restructures slower than it sheds monomers to the solution, $k_{2}+k_{-2}\ll k_{-1}$ and $k_{a}=K_{1}k_{2}$. The saturating *R*(*C*_*Aβ*40_) branch for bexarotene fibrils at *C*_*Aβ*40_, for which *R* of normal fibrils increases linearly with *C*_*Aβ*40_ (Fig. 5*E*), announces that *k*_1_*C*_*Aβ*40_ supersedes $k_{-1}+k_{2}+k_{-2}$ at much lower *C*_*Aβ*40_ than during growth of normal fibrils owing to disparate values of the rate constants *k*_*i*_. The discrepancy between the rate constants for bexarotene and normal fibrils argues that the unique structure of the bexarotene fibrils (Figs. 3 and 4) dictates an intermediate complex with distinct properties that may relate to a unique arrangement of the constituent peptide chains.

The response of the rates of growth of bexarotene fibrils to the addition of urea concurs with the conclusion of distinct properties of the complex at their tips. Owing to its favorable interaction with the amide groups of the peptide backbones (58), urea denatures most proteins (59) and weakens the contacts that support the structures of amyloid fibrils (60). Urea impacts the growth of normal fibrils in two ways. It boosts the solubility by stabilizing the solute peptide chains and accelerates fibril growth by impairing the contacts that uphold the complex at the fibril tips (40). For bexarotene fibrils, the narrow range of the ascending branch of the *R*(*C*_*Aβ*40_) correlation precludes inferences on how urea affects fibril solubility. In the saturated branch of *R*(*C*_*Aβ*40_), urea does not appear to affect the fibril growth rate *R*_max_ = *ak*_2_. The constant *k*_2_ characterizes the rate of restructuring of a peptide chain from the intermediate complex to the conformation in the fibril bulk. Its insensitivity to urea for bexarotene fibrils advocates that their intermediate complex primarily relies on bonds that weakly respond to urea (61) (Table S2), in sharp contradistinction to normal fibrils (40).

### The fibrils with bexarotene-engineered structure show reduced neurotoxicity

**Figure 6:**
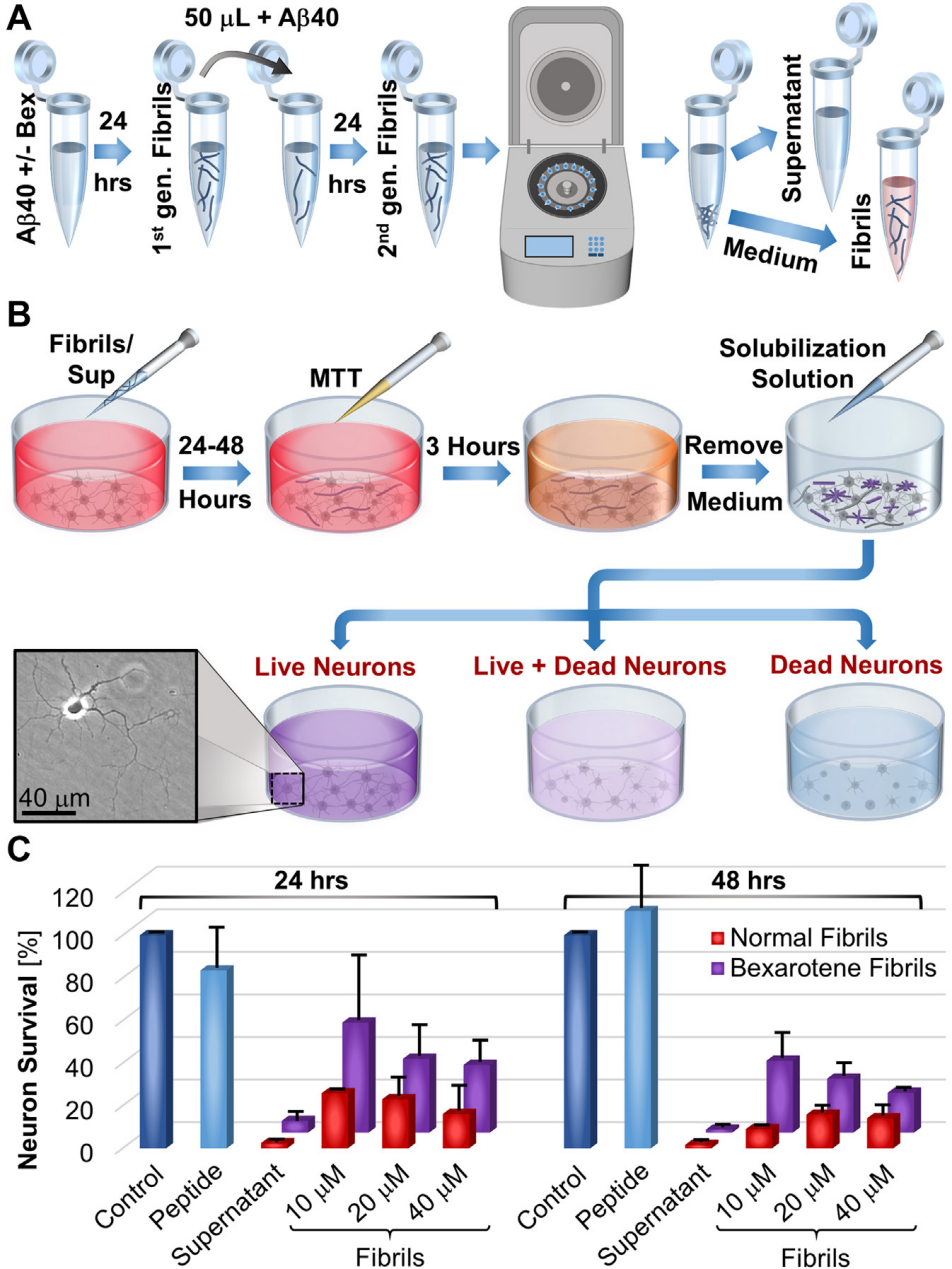
**Neurotoxicity of normal and bexarotene fibrils.***A*, schematic of the isolation of normal and bexarotene fibrils from their respective supernatants. *B*, schematic illustration of the MTT assay to measure the toxicity of Aβ40 fibrils to primary rat hippocampal neurons. An optical micrograph of a live neuron is shown. *C*, neuron survival fractions after exposure to solutions of Aβ40 peptide, supernatants separated from the respective fibrils, and fibrils at three total peptide concentrations for 24 and 48 h. *Red bars* indicate normal fibrils and supernatant, and *purple bars* indicate bexarotene fibrils and supernatant. Control (*navy*) is only neurobasal medium. The volume of the added supernatant was equal to that for the 20 μM fibril suspension. The determinations were performed three times. Error bars indicate the standard deviations from the average fractions of surviving neurons. The parameters of the ANOVA tests of the similarity between the toxicities of normal and bexarotene fibrils and their supernatants are presented in Tables S3 and S4. Aβ, amyloid β; Aβ40, 40-residue Aβ isoform; MTT, 3-(4,5-dimethylthiazol-2-yl)-2,5-diphenyltetrazolium bromide; Sup, supernatant.

Neurotoxicity is a cardinal feature of amyloid fibrils, in which the known Aβ40 fibril polymorphs severely diverge (32, 49, 50). We test whether the distinct structure of the fibrils nucleated in the presence of bexarotene may motivate higher toxicity to neurons, which would, at least partially, negate the benefit of their slow growth. We quantify the survival of primary rat hippocampal neurons after exposure for 24 and 48 h to five Aβ40 specimens: freshly prepared Aβ40 solution, fibrils grown in the presence and absence of bexarotene, and the respective fibrils’ supernatants (Fig. 6*A*). We select the concentration of the Aβ40 solution, 2 μM, near the solubility of normal fibrils, 0.44 μM (40, 41), to approximately match the concentration of the supernatants and to minimize the probability of fibrillization. We prepare fibrils analogously to the fibrils used in AFM growth experiments and structure determinations and separate them from their respective supernatants by centrifugation (Fig. 6*A*). The fibril equilibration time, 24 h (Fig. 6*A*), is substantially longer than the time, 10 to 14 h, over which fibrillization ceases as the solution concentration approaches the solubility (Fig. 1*D*). The selected long equilibration time allows the fibrils to capture most of the Aβ40 amounts that exceed the solubilities of normal and bexarotene fibrils. We suspended the fibrils in the neuron culture medium and treat the neurons with aliquots that contain fibril mass sufficient to bring the final Aβ40 concentration to ca. 10, 20, and 40 μM. To quantify neuron survival, we add to the treated neurons 3-(4,5-dimethylthiazol-2-yl)-2,5-diphenyltetrazolium bromide (MTT), a yellow dye (32). Mitochondrial dehydrogenases produced from viable neurons reduce MTT to purple formazan, which is insoluble in the culture medium. After 3 h incubation with MTT, we removed the cell culture medium to eliminate its absorbance and added a buffer that dissolves the formazan crystals into a purple-colored solution (Fig. 6*B*). The absorbance of this solution at 570 nm quantifies the formazan concentration and the fraction of live neurons (Fig. 6*B*).

The measurements of the formazan absorbance demonstrate that primary rat hippocampal neurons are largely indifferent to Aβ40 peptide solutions (Fig. 6*C*). By contrast, both normal and bexarotene fibrils kill the neurons (Fig. 6*C*). Surprisingly, the supernatants kill neurons more potently than the fibrils themselves. The greater toxicity of the supernatants may be due to Aβ40 peptide oligomers (27, 29, 31, 56, 62, 63, 64, 65, 66) present in the solution after fibrillization ceases or released during centrifugation. Notably, the fibrils added to the rat neurons were from freshly solubilized centrifugation pellets and did not contain any supernatant. Thus, the concentration of bexarotene, which may promote neurogenesis (67), in the fibril suspension is negligible. More neurons survive after 48 h exposure to bexarotene-generated fibrils than to normal fibrils. The distinctions between the toxicities of two types of amyloids after 24 h exposure to fibrils and after exposure to supernatants for both 24 and 48 h are not statistically significant (Fig. 6*C*, Tables S3 and S4). Importantly, these statistics declare that amyloids with bexarotene-sanctioned structure are not more toxic to primary rat hippocampal neurons than their normal equivalents.

## Conclusions

The responses to bexarotene of the structure and growth rate of Aβ40 fibrils reveal that a drug may suppress fibrillization even if it does not bind to fibrils. We demonstrate that compounds that have a potential as drugs may engineer a distinct fibril structure, whose growth stalls owing to a unique intermediate state for incorporation of solute peptides. Suppression of fibril growth by drug-induced polymorph transformation may present a superior mode of drug action: since the fibril structure is selected during nucleation and persists during growth, fibrils with drug-enforced structure grow more slowly than normal fibrils even in the absence of the drug in the environment that hosts the fibrils. We show that a drug-enforced fibril structure may carry a supplementary advantage of lower neurotoxicity. The reengineering of the fibril structure by bexarotene exposes the rich variety of fibrillization pathways that may be targeted by strategies to suppress and reverse amyloid fibrillization as a route to treating Alzheimer’s and other neuropathies.

## Experimental procedures

A summary of experimental procedures is provided later with additional details included in the Supporting information.

### Solution preparation

Fibril growth buffer was prepared by dissolving sodium phosphate monohydrate (Mallinckrodt) in deionized water to a final concentration of 40 mM and adjusting pH to 7.4 using NaOH. Bexarotene stock solution was prepared by dissolving bexarotene (Sigma–Aldrich) in dimethyl sulfoxide (Sigma–Aldrich) to a final concentration of 0.2 mM.

### Aβ40 expression and purification

We followed published methods to express and purify Aβ40 (68). We used Western blot and LC–MS to confirm the identity of the purified peptides (Fig. S1, *A–D*).

### ThT fluorescence assay

The stock solution of ThT was prepared by dissolving ThT (Sigma–Aldrich) in ethanol (Decon Labs) and filtering through 0.22 mm polyethersulfone syringe filter. To monitor the fibril formation, Aβ40 peptides were diluted to 50 μM in fibril growth buffer in a black 96-well plate with clear bottom. For control experiment, ammonium acetate solution in a volume equivalent to the peptides volume was added to the growth buffer instead of Aβ40 peptides. To monitor the effect of bexarotene on Aβ40 fibrillization, bexarotene was added to diluted Aβ40 solution at final desired concentrations. ThT then was added to the Aβ40, Aβ40-bexarotene, and control solutions to a final concentration of 35 μM. The ThT fluorescence signal was measured at 37 ^o^C (with mixing every 5 min) every 15 min with excitation and emission at 442 and 488 nm, respectively, for a period of 15 h using SpectraMax Gemini EM Microplate Reader (Molecular Devices). The sigmoid curves were obtained by subtracting the absorbance of the control and normalizing all the data points to their maximum value.

### Complexation of Aβ40 with bexarotene

To test whether Aβ40 peptides form complex with bexarotene, Aβ40 peptide solution (in ammonium acetate) with initial volume of 600 μl and concentration of 156 μM was titrated with bexarotene by adding 20 μl aliquots of a solution of 142 μM bexarotene in ammonium acetate. This addition was repeated 20 times. The absorbance of the Aβ40–bexarotene mixture at 280 nm was compared with the sum of the individual absorbance of bexarotene and Aβ40 peptides at the corresponding concentrations. The agreement of the two absorbance values at all concentrations indicates that no complex was formed (Fig. S2, *C* and *D*).

### Preparation of normal and bexarotene fibrils

Bexarotene fibrils grew at the same condition as normal fibrils (41). These first-generation fibrils then were used as seeds for the second-generation fibrils. The stock Aβ40 peptides were diluted to 50 μM in the growth buffer with 10% v/v of the first-generation fibrils and subjected to the same growth conditions as the first-generation fibrils for another 24 h.

### Time-resolved *in situ* AFM

To prepare samples for AFM growth rate measurements, 2 μl of second-generation normal (or bexarotene) fibrils was added to the fibril growth buffer. Urea (Sigma–Aldrich) was also added to the solution, to a final concentration of 1 M, if needed based on the experiments. The diluted fibril solution then was sonicated (6 W output) for 2 min with 15 s intervals on ice and was kept at 27 °C for at least 15 min to equilibrate the temperature. Then Aβ40 peptides were added to the fibril solution at a desired final concentration, and the total volume of the solution was adjusted to 1 ml.

Multimode atomic force microscope (Nanoscope VIII or IV; Bruker) in tapping mode was used to monitor the growth of fibrils. To collect images, 500 μl of the prepared sample was injected into the AFM liquid cell over freshly cut mica (Ted Pella, Inc) attached to a 15 mm metal disk (Ted Pella, Inc), and to avoid any leakage, an O-ring was inserted firmly to the liquid cell. The temperature in the liquid cell reached equilibrium of 27.0 ± 0.1 °C within 15 min, higher than room temperature (ca. 22 °C), because of heating by the AFM scanner and laser (69, 70, 71). Height, amplitude, and phase images were collected in image sizes ranged from 2 μm × 2 μm to 8 μm × 8 μm, and scan rates range between 3 and 3.5 s^−1^ in most images.

To study the effect of bexarotene on normal and bexarotene fibril growth rates (Figs. 2*D* and 5*D*), first, a fibril suspension at 3 μM Aβ40 peptide concentration was prepared as described previously, without bexarotene, and the fibril growth was monitored with AFM in tapping mode for 15 to 30 min. Then the solution in the liquid cell was replaced with a fresh solution containing the same concentration of Aβ40 peptides and desired concentration of bexarotene, with a volume adjusted to 1 ml by the fibril growth buffer. Image collection was continued with the new solution to measure the growth rates of Aβ40 normal and bexarotene fibrils after addition of bexarotene at different concentrations.

A sequence of 10 to 16 images were collected to measure the displacement of each fibril end from an immobile reference point using Nanoscope analysis or ImageJ (National Institutes of Health) and determine the growth rates. The reported growth rates at each Aβ40 concentrations were represented by the average of 20 to 40 fibril end growth rate measurements. The correlation between the growth rates of the opposing ends of bexarotene fibrils suggests asymmetric growth (Fig. S5, *A–F*), in contrast to the symmetric growth of normal fibril ends (41).

To obtain the thickness distribution, the heights of 68 and 60 normal and bexarotene, respectively, fibrils were measured. The thickness of each fibril was determined by the average height of at up to 10 cross sections along the fibril length. The thickness remains unchanged along the fibril axis, and growth rates of normal and bexarotene fibrils have no correlations with the fibril thickness (Fig. S6, *A–C*).

### Cryo-EM imaging

To prepare normal and bexarotene fibril samples for cryo-EM, 20 μl of second-generation fibrils was added to the fibril growth buffer at a final volume of 500 μl and sonicated (6 W output) for 1 min with 15 s intervals on ice. Aβ40 peptide solution was added to the fibril suspension to a final concentration of 50 μM. The sample was sonicated in a water bath sonicator (Cole-Parmer Ultrasonic Bath; Cole-Parmer) for 1 min to eliminate fibril clumping. Images were collected with a Titan Krios G3i electron microscope (Thermo Fisher Scientific) operating at 300 keV and equipped with a GIF Quantum LS energy filter (Gatan) and a K3 direct electron detector camera (Gatan). We used 20 eV energy slit width during data acquisition. Total electron dose/image was ∼40 electrons/Å^2^. Image pixel size was 1.1 Å on the specimen scale for normal fibrils and 0.8 Å for bexarotene fibrils.

The crossover distances and fibril widths were measured from 59 micrographs for normal fibrils and 67 micrographs for bexarotene fibrils using Fiji software (72). Several individual measurements were taken from each micrograph. In total, 227 crossover distances, 228 fibril widths at the widest points between crossovers, and 292 widths at the crossovers were measured for normal fibrils; 247 crossover distances, 262 fibril widths at the widest points between crossovers, and 291 widths at the crossovers for bexarotene fibrils.

### Neurotoxicity assay

Primary embryonic rat hippocampal neurons (Sprague–Dawley embryonic day 18 rats; Thermo Fisher Scientific) were cultured in neurobasal medium (Thermo Fisher Scientific), supplemented with 2% v/v B-27 supplement (Thermo Fisher Scientific), 0.5 mM glutamine (Thermo Fisher Scientific), 25 μM l-glutamate (Fisher Scientific) (only up to day 4 of incubation), and 1% v/v antibiotic–antimycotic (Sigma–Aldrich). The cells were seeded in a 96-well plate coated with 50 μg/ml poly-d-lysine (Thermo Fisher Scientific) to achieve 3 × 10^4^ cells per well and incubated at 37 °C in a humidified atmosphere of 5% CO_2_ for 7 days (the medium was replaced every 3 days) before exposure to Aβ40 fibrils. Images of cultured neurons were obtained (EVOS M7000 Florescence Microscopy; Thermo Fisher Scientific) to ensure that neural network was formed.

### Statistical tests for similarity between groups of data on fibril morphology and toxicity

To test if the growth rates of the normal and bexarotene fibrils in the absence and presence of bexarotene at the specified concentrations (Figs. 2*D* and 5*D*) are statistically identical, one-way ANOVA was employed, which compares the variance between each group to the variance within each group.

For normal fibrils, the *F* value is 0.32, smaller than critical *F* value 2.69 resulted from four groups of 34, 29, 27, and 24 individual measurements with a 95% confidence interval. The *p* value is 0.81, greater than the α value of 0.05. The *F* value and *p* value indicate the null hypothesis is true, which means the mean values are the same for all independent groups. Therefore, the growth rates of normal fibrils are not affected by bexarotene at all indicated concentrations.

We also performed Kruskal–Wallis test for the data in Figure 2*D* (Table S5). The obtained *p* value of 0.75 is greater than the α value of 0.05 and suggests that the null hypothesis that the mean ranks of the groups are the same is true. This result advocates that bexarotene does not affect the growth rates of normal fibrils, consistent with ANOVA test results.

For bexarotene fibrils, the *F* value from two groups of 24 and 36 individual measurements is 0.19, smaller than critical *F* value of 4.01, and the *p* value is 0.66, greater than the α value of 0.05 (95% confidence interval), suggesting that the two groups are statistically identical. This ratifies that bexarotene does not affect the growth rates of bexarotene fibrils.

The same one-way ANOVA tests were performed for normal and bexarotene fibril thicknesses measured by AFM, fibril widths, and widths at the crossovers (Table S3). In all cases, *F* values are greater than critical *F* values, and *p* values are smaller than the α value of 0.05 (95% confidence interval), rejecting the null hypothesis, which suggests the two groups are statistically different.

The two-way ANOVA test was performed for the neurotoxicity measurements. Two categories of normal and bexarotene fibrils have three concentrations of fibrils (10, 20, and 40 mM) each (Table S4). The *p* value for normal and bexarotene fibril categories is greater than α value of 0.05 for 24 h treatment and smaller than the α value of 0.05 for 48 h treatment, which means the neurotoxicity of normal and bexarotene fibrils are statistically identical after 24 h, whereas, different after 48 h. The *p* values for comparisons between fibril concentrations are greater than the α value of 0.05 for both 24 and 48 h treatment, suggesting that increasing fibril concentration does not affect the neurotoxicity of fibrils. Also, the *p* values for interaction between two categories are greater than the α value of 0.05 for both 24 and 48 h treatment, which means null hypothesis cannot be rejected and there is no correlation between neurotoxicity of normal and bexarotene fibrils and fibril concentrations.

## Data availability

The datasets generated during and analyzed during the current study are available from the corresponding author upon reasonable request.

## Supporting information

This article contains Supporting information (31, 33, 40, 41, 47, 68).

## Conflict of interest

The authors declare that they have no conflicts of interest with the contents of this article.
