# Supplementary material for: Suppression of amyloid-β fibril growth by drug-engineered polymorph transformation
Source: J Biol Chem. 2022 Nov 2;298(12):102662. doi: 10.1016/j.jbc.2022.102662 (PMC9720346; doi:10.1016/j.jbc.2022.102662)
Supplement: Supplemental Figures S1–S6 and Tables S1–S5 [file mmc1.docx]

Supplementary Information for

Suppression of amyloid β fibril growth by drug-engineered polymorph transformation

Sima Mafimoghaddam, Yuechuan Xu, Michael B. Sherman, Elena V. Orlova, Prashant Karki, Mehmet A. Orman, and Peter G. Vekilov

# Materials and methods

**Solution Preparation.** A stock solution of 100 mg mL^-1^ ampicillin was prepared by dissolving ampicillin sodium salt (Fisher Scientific) in deionized (DI) water produced by a reverse osmosis ion-exchange system (Rios-8 Proguard 2, MilliQ Q-guard system, MilliporeSigma). LB-ampicillin growth medium was prepared by dissolving LB (Sigma Aldrich) in DI water to a final concentration of 20 g L^-1^ and adding ampicillin stock solution to the final concentration of 100 μg mL^-1^ in the LB medium. To prepare IPTG (isopropyl β-D-1-thiogalactopyranoside, Sigma Aldrich) stock solution, IPTG was dissolved in DI water to a final concentration of 100 mM. Both ampicillin and IPTG stock solutions were filter sterilized using 0.22 μm PES (polyethersulfone membrane) syringe filter (CELLTREAT) and aliquots were stored at -20 °C.

EDTA (Sigma Aldrich) and Tris (Sigma Aldrich) were dissolved in DI water to the final concentrations of 1 mM and 10 mM, respectively, to prepare the sonication buffer and pH was adjusted to 8.0 using HCl (Sigma Aldrich). Binding buffer was prepared by adding urea (Sigma Aldrich) to the sonication buffer to a concentration of 8 M, and adjusting pH to 8.0. To prepare elution buffer, NaCl (Sigma Aldrich) was dissolved in binding buffer to the desired concentrations (50, 75, 100 and 500 mM), and pH was adjusted to 8.0.

Ammonium acetate (Sigma Aldrich) was dissolved in DI water to a concentration of 50 mM and pH of the solution was adjusted to 8.5 using NaOH (Sigma Aldrich) to prepare the equilibration and elution buffer for size exclusion chromatography (SEC).

Fibril growth buffer was prepared by dissolving sodium phosphate monohydrate (Mallinckrodt) in DI water to a final concentration of 40 mM and adjusting pH to 7.4 using NaOH.

Bexarotene stock solution was prepared by dissolving bexarotene (Sigma Aldrich) in DMSO (dimethyl sulfoxide, Sigma Aldrich) to a final concentration of 0.2 mM.

**Aβ40 expression and purification.** To express Aβ40**,** following published methods(1), we used QIAprep Spin Miniprep Kit (QIAGEN) to extract the pET-Sac-Abeta(M1-40) plasmid (Addgene, MDAEFRHDSGYEVHH QKLVFFAEDVGSNKGAIIGLMVGGVVIA). The plasmid was transformed into *E.coli* BL21(DE3) (provided by Dr. Patrick Cirino at University of Houston) by electroporation. The transformed *E. coli* grew on an LB agar plate at 37 °C overnight and was then inoculated into 10 mL of LB-ampicillin growth medium. The culture was incubated in a shaker (MaxQ 5000, Barnstead Lab-Line) at 37 °C and 250 rpm until the optical density at 600 nm (OD_600_), measured by NOVOstar Plate Reader (BMG Labetch), reached at least 2.0. Then the culture was diluted 100-fold into LB-ampicillin growth medium and allowed to grow further in the same incubation condition. Once the OD_600_ reached 0.5- 0.6, IPTG to a final concentration of 100 μM was added and the culture was allowed to grow for about four hours in the same incubation condition until the final OD_600_ reached around 1.0. To harvest the cells, the culture was centrifuged (Sorvall Legend X1R Centrifuge, Thermo Fisher) for 20 minutes at 4 °C and 5000 rpm and the supernatant was discarded.

The harvested cells were re-suspended in 20 mL of ice-cold sonication buffer, sonicated (Misonix Sonicator XL-2000, Qsonica,LLC.) at 10 W output on ice for total of two minutes with 30-second intervals, and centrifuged (Avanti J-E Ultra Centrifuge, Beckman Coulter) for 10 minutes at 4 °C and 18000 × g. Then the supernatant was discarded and the same procedure was repeated with the sonication buffer for another round. In the third round, ice-cold binding buffer was added to the cells and the same sonication and centrifugation procedures were performed. The supernatant of the third round was retained, filtered with 0.45 μm PES syringe filter (VWR International), and diluted in 20 mL of ice-cold binding buffer.

Pre-chilled Q Sepharose Fast Flow resin (GE Healthcare Lifescience) was equilibrated in ice-cold sonication buffer. Then the diluted lysate solution was mixed with the resin, 4:1 lysate to resin ratio, on a STD Vortex Mixer (Fisher Scientific) for 15 minutes at 4 ^o^C, then centrifuged for 15 minutes at 4 ^o^C and 2500 × g (Sorvall Legend X1R Centrifuge, Thermo Fisher), and the supernatant was discarded. The resin was eluted by elution buffer with 50, 75, 100, and 500 mM NaCl, respectively, following the same mixing and centrifugation procedures. The fractions with most Aβ40 were collected from 50 and 75 mM NaCl eluates for further purification steps.

To concentrate, the collected fractions were filtered with 0.45 μm PES syringe filter, moved to a 30 kDa MWCO (molecular weight cut off) Ultra Centrifugal Filtration Unit (Amicon), and centrifuged at 4 ^o^C and 4000 × g until retentate was adjusted to 500 μL. The filtrate was subsequently moved to a 3 kDa MWCO filtration unit and the same centrifugation procedure was performed. Both 3 and 30 kDa MWCO filters were equilibrated with sonication buffer before the addition of Aβ40 fractions. Retentates from the two steps were sent through SEC using Superdex 75 16/20 XK column (GE Healthcare Lifescience) in ÄKTA Pure System (GE Healthcare Lifescience) at 4 ^o^C , and eluted at 0.7 mL min^-1^ with the ammonium acetate buffer. The isolated Aβ40 peptides were collected in fractions of 0.7 mL based on the absorbance peak at 280 nm from the chromatogram (Fig. S1A). The concentration of peptides then was determined by measuring absorbance at 280 nm (NanoDrop Lite, Thermo Fisher Scientific) using ε_280_ = 1490 M^-1^ cm^-1^, and adjusted to 100-150 µM based on productivity using 3 kDa MWCO filtration unit. At last, purified Aβ40 peptides were filtered using the 0.45 µm PES syringe filter, aliquoted based on desired working concentrations, froze with liquid nitrogen, and stored in -80 ^o^C.

**Identification of Aβ40.** Western blot and liquid chromatography–mass spectrometry (LC-MS) confirmed the identity of the purified peptides (Fig. S1, A to D). To perform western blot, we followed the BIO-RAD Western Blot protocol. Primary amyloid beta polyclonal antibody rabbit (Fisher Scientific) was used to bind with Aβ40 and goat anti-rabbit IgG secondary antibody HRP (Fisher Scientific) was used to detect binding of primary antibody. The binding of secondary antibody was tested with 1-step Ultra TMB-Blotting HRP Substrate solution (Fisher Scientific). A colored fragment appeared on the gel which confirms the presence of Aβ40 peptide (Fig. S1D)

LC-MS analysis was conducted using a Shimadzu Prominence LC-20AD XR UPLC system interfaced to a Shimadzu Ion Trap – Time of Flight (IT-ToF) Mass Spectrometer (MS) through an Electrospray Ionization (ESI) source. Separations were achieved using a 1.0 mm ID x 150 mm, 2.7 micron particle size Ascentis Express Peptide ES-C18 column. The column was operated at a flow rate of 0.15 mL/min with mobile phase A: 0.1% (v/v) formic acid in water and mobile phase B: 0.1% (v/v) formic acid in acetonitrile. The LC gradient for the analytical separation was operated as follows; 5% B (IC), 5% B to 30%B (0.0 min to 12.5 min), 30%B to 50%B (12.5 min to 14.5 min), and 50%B to 95%B (14.5 min to 16.0 min). MS spectra were acquired over the range of m/z 300 to m/z 1200 (Fig. S1B). The open source mass spectrometry tool mMASS was used to assist with data analysis(2).

**Thioflavin T (ThT) fluorescence assay.** The stock solution of ThT was prepared by dissolving ThT (Sigma Aldrich) in ethanol (Decon Labs), and filtering through 0.22 μm PES syringe filter. The concentration was determined by measuring the absorbance at 416 nm using the extinction coefficient of 26.6 mM^-1^ cm^-1^. To monitor the fibril formation, Aβ40 peptides were diluted to 50 μM in fibril growth buffer in a black 96-well plate with clear bottom. For control experiment, ammonium acetate solution in a volume equivalent to the peptides volume was added to the growth buffer instead of Aβ40 peptides. To monitor the effect of bexarotene on Aβ40 fibrillization, bexarotene was added to diluted Aβ40 solution at final desired concentrations. ThT then was added to the Aβ40, Aβ40-bexarotene, and control solutions to a final concentration of 35 μM. The ThT fluorescence signal was measured at 37 ^o^C (with mixing every 5 minutes) every 15 minutes with excitation and emission at 442 nm and 488 nm, respectively, for a time period of 15 hours using SpectraMax Gemini EM Microplate Reader (Molecular Devices). The sigmoid curves were obtained by subtracting the absorbance of the control and normalizing all the data points to their maximum value.

Alternatively, the delayed increase of ThT fluorescence intensity in the presence of bexarotene may be due to binding of bexarotene to the fibrils, which displaces ThT and lowers the signal from the fibril-bound ThT. This scenario would enforce a ratio between the fluorescence intensity in the presence and absence of bexarotene that is constant in time and decreases with higher bexarotene concentration. The experimentally observed ratio varies between zero and unity as fibrillization proceeds, rejecting the competitive binding artefact hypothesis.

This conclusion is consistent with our ThT and AFM results, which collectively demonstrate that bexarotene interacts with pre-nucleation oligomers only. The prenucleation oligomers, as suggested by recent MD simulations (3), structure distinctly from mature fibrils. ThT is unlikely to bind to such oligomers. Correspondingly, ThT fluorescence intensity does not increase and stays at zero during the nucleation delay stage. Furthermore, the AFM results indicate that bexarotene does not interact with growing fibrils, hence, ThT could not compete with it for binding to the fibrils.

We fit the data on the increase of ThT fluorescence intensity in the absence and in the presence of several bexarotene concentrations (Fig. 1D) with the models that comprise AmyloFit (4). We found that the simplest model, Nucleation Elongation, Unseeded, fits the data poorly. Several models, Secondary Nucleation Dominated, Unseeded; Fragmentation Dominated, Unseeded; Fragmentation and Secondary Nucleation, Unseeded; and Multi-step Secondary Nucleation Dominated, Unseeded, provided reasonable and about equally good fits. The results provided no guidance on which model and parameter set to choose.

**Complexation of Aβ40 with bexarotene**. To determine the extinction coefficient of bexarotene at 280 nm, we measured the absorbance of bexarotene at 14 gradually decreasing concentrations (Fig. S2A). The stock solution of bexarotene (0.2 mM) was diluted in ammonium acetate to achieve the desired concentrations. Then, the correlation between absorbance and bexarotene concentration was plotted and the extinction coefficient was obtained from the slope of the best linear regression fit (ε_280_ = 10370 ± 83 M^-1^ cm^-1^).

To test whether Aβ40 peptides form complex with bexarotene, Aβ40 peptide solution (in ammonium acetate) with initial volume of 600 μL and concentration of 156 μM was titrated with bexarotene by adding 20 μL aliquots of a solution of 142 μM bexarotene in ammonium acetate. This addition was repeated 20 times, and at each step, a spectrum was collected using DU 800 Spectrophotometer (Beckman Coulter). The absorbance of the Aβ40-bexarotene mixture at 280 nm was compared to the sum of the individual absorbance of bexarotene and Aβ40 peptides at the corresponding concentrations. The agreement of the two absorbance values at all concentrations indicates that no complex was formed (Fig. S2, C and D).

**Preparation of normal and bexarotene fibrils.** To prepare normal first-generation fibrils, Aβ40 peptides were diluted to 100 μM in the fibril growth buffer at a final volume of 500 μL, and continuously stirred at 37 ^o^C and 300 rpm on a table Inkubator 1000 (Heidolph) for 24 hours. For bexarotene fibrils (first-generation), Aβ40 peptides were diluted to 75 μM in the fibril growth buffer and bexarotene stock solution was added to Aβ40 peptides to a final concentration of 75 μM (1:1 peptide to drug ratio). Bexarotene fibrils grew at the same condition as normal fibrils. These first-generation fibrils then were used as seeds for the second-generation fibrils. The stock Aβ40 peptides were diluted to 50 μM in the growth buffer with 10% v/v of the first-generation fibrils, and subjected to the same growth conditions as the first-generation fibrils for another 24 hours.

Bexarotene was dissolved in DMSO prior to its addition to the Aβ40 solutions. Evidence in the literature indicates that pure DMSO supports monomeric Aβ and impedes the formation of β−sheets(5). Importantly, when added to aqueous solvents at concentrations up to 10 % DMSO may be insufficiently potent to disrupt secondary structure and does not affect amyloid formation(5). Further evidence implies that DMSO concentrations around 1 % have no effect on protein native structure and concentrations in the range 50 to 100% are needed to disrupt secondary protein structure(6). Measurements of ThT fluorescence demonstrate that DMSO at 1% has no effect on the kinetics of fibrillization of Aβ42 ref. (7). In our experiments the DMSO concentrations in the solutions from which Aβ40 fibrils grow were always equal or less than 1 % and bolster our contention that DMSO does not affect the observed responses to bexarotene addition.

**Time resolved *in situ* atomic force microscopy (AFM).** To prepare samples for AFM growth rate measurements, 2 μL of second-generation normal (or bexarotene) fibrils was added to the fibril growth buffer. Urea (Sigma Aldrich) was also added to the solution, to a final concentration of 1 M, if needed based on the experiments. The diluted fibrils solution then was sonicated (6 W output) for 2 minutes with 15-second intervals on ice, and was kept at 27 °C for at least 15 minutes to equilibrate the temperature. Then Aβ40 peptides were added to the fibril solution at a desired final concentration and the total volume of the solution was adjusted to 1 mL.

Multimode atomic force microscope (Nanoscope VIII or IV, Bruker) in tapping mode was used to monitor the growth of fibrils. Images were collected using TR800PSA probes (Silicon nitride, Cr/Au coated 5/30, 0.15 N/m spring constant, Asylum Research) or SNL-10 probes (Silicon nitride, reflective gold coated, 0.24 spring constant, Bruker). To collect images with AFM, 500 μL of the prepared sample was injected into the AFM liquid cell over freshly cut mica (Ted Pella Inc.) attached to a 15 mm metal disk (Ted Pella Inc.) and to avoid any leakage, an O-ring was inserted firmly to the liquid cell. The temperature in the liquid cell reached equilibrium of 27.0 ± 0.1°C within 15 minutes, higher than room temperature (ca. 22°C), due to heating by the AFM scanner and laser.

Height, amplitude, and phase images were collected in image sizes ranged from 2 µm × 2 µm to 8 µm × 8 µm, and scan rates between 3 and 3.5 s^-1^ in most images. Since images contained 256 scan lines at 0 angle, collection of one image took approximately 1.5 to 4 minutes. To illustrate the fibril growth, amplitude images were employed in which the image intensity corresponds to the deviation of the tip response frequency from the driving frequency due to interaction with the fibrils. Also, the height images were used to determine the morphological characteristics of a fibril such as thickness.

To study the effect of bexarotene on normal and bexarotene fibrils growth rates (Figs. 2D and 5D), first, a fibril suspension at 3 µM Aβ40 peptide concentration was prepared as described above, without bexarotene, and the fibril growth was monitored with AFM in tapping mode for 15 to 30 minutes. Then the solution in the liquid cell was replaced with a fresh solution containing the same concentration of Aβ40 peptides and desired concentration of bexarotene, with a volume adjusted to 1 mL by the fibril growth buffer. Image collection was continued with the new solution to measure the growth rates of Aβ40 normal and bexarotene fibrils after addition of bexarotene at different concentrations.

A sequence of 10-16 images were collected to measure the displacement of each fibril end from an immobile reference point using Nanoscope analysis or ImageJ and determine the growth rates. The reported growth rates at each Aβ40 concentrations were represented by the average of 20 to 40 fibril ends growth rate measurements. The correlation between the growth rates of the opposing ends of bexarotene fibrils suggests asymmetric growth (Fig. S5, A to F), in contrast to the symmetric growth of normal fibril ends(8).

To obtain the thickness distribution, 60 to 70 height measurements were performed for each normal and bexarotene fibrils. The thickness of each fibril was determined by the average height of at least three cross sections along the fibril length. The thickness remains unchanged along the fibril axis and growth rates of normal and bexarotene fibrils have no correlations with the fibril thickness^3^ (Fig. S6, A to C).

**Cryogenic electron microscopy (cryo-EM) imaging.** To prepare normal and bexarotene fibrils sample for cryo-EM, 20 μL of second-generation fibrils was added to the fibril growth buffer at a final volume of 500 μL and sonicated (6 W output) for 1 minute with 15-second intervals on ice. Aβ40 peptide solution was added to the fibrils suspension to a final concentration of 50 μM. The sample was sonicated in a water bath sonicator (Cole-Parmer Ultrasonic Bath, Cole-Parmer) for one minute to eliminate fibril clumping.

Normal or bexarotene Aβ40 fibrils were vitrified using Leica EM-GP2® plunger (Leica Microsystems) as previously described(9) on carbon holey film (R2x1 Quantifoil®; Micro Tools GmbH, Jena, Germany) grids. Briefly, 4 µl of fibril suspensions were applied to the grids pre-cleaned in a Solarus 950 plasma cleaner (Gatan), blotted with filter paper, and plunged into liquid ethane. The frozen grids were stored under liquid nitrogen until used for microscopy. Grids were transferred into a Titan Krios G3i electron microscope (ThermoFisher Scientific) operating at 300 keV and equipped with a GIF Quantum LS energy filter (Gatan) and a K3 direct electron detector camera (Gatan). We used 20 eV energy slit width during data acquisition. Total electron dose/image was ~40 electrons/Å2. Image pixel size was 1.1 Å on the specimen scale for normal fibrils, and 0.8 Å for bexarotene fibrils.

The crossover distances and fibrils widths were measured from 59 micrographs for normal fibrils, and 67 micrographs for bexarotene fibrils using Fiji software. Several individual measurements were taken from each micrographs. In total, 227 crossover distances, 228 fibril widths at the widest points between crossovers, and 292 widths at the crossovers were measured for normal fibrils; 247 crossover distances, 262 fibril widths at the widest points between crossovers, and 291 widths at the crossovers for bexarotene fibrils.

We report the average crossover distance, width, and width at crossover for normal and bexarotene fibrils (Table S1) with their respective standard deviations. Since the crossover distance of bexarotene fibrils is not uniformly distributed (Fig. 4E), we divided bexarotene fibrils into two subgroups, short (< 40 nm) and long (> 80 nm) crossovers, and we record the average crossover distance of each subgroup separately.

**Neurotoxicity assay.** Primary embryonic rat hippocampal neurons (Sprague Dawley embryonic day-18 rats, Thermo Fisher Scientific) were cultured in neurobasal medium (Thermo Fisher Scientific), supplemented with 2% v/v B-27 supplement (Thermo Fisher Scientific), 0.5 mM glutamine (Thermo Fisher Scientific), 25 μM L-glutamate (Fisher Scientific) (only up to day 4 of incubation), and 1% v/v antibiotic-antimycotic (Sigma Aldrich). The cells were seeded in a 96-well plate coated with 50 μg/mL poly-D-lysine (Thermo Fisher) to achieve 3×10^4^ cells per well, and incubated at 37°C in a humidified atmosphere of 5% CO2 for 7 days (the medium was replaced every 3 days) before exposure to Aβ40 fibrils. Images of cultured neurons were obtained (EVOS M7000 Florescence Microscopy, Thermo Fisher) to ensure neural network was formed.

Normal and bexarotene (second-generation) fibrils in growth buffer were pelleted by centrifugation for 30 min at 21000 ×g using Sorvall Legend Micro 21 Centrifuge (Thermo Fisher Scientific). The supernatant was retained for control experiment and replaced with neurobasal complete medium. Prior to addition to cells, fibril suspensions were sterilized by UV irradiation, diluted in neurobasal culture medium to achieve the desired Aβ40 concentrations, and sonicated in a water bath sonicator for two minutes. The neurons were treated with 2 μM Aβ40 peptide solution, normal and bexarotene fibrils at 10, 20 and 40 μM concentrations of Aβ40, and retained supernatants from both normal and bexarotene fibril suspensions in a volume equal to that for 20 μM fibril suspension for 24 and 48 hours. For control, only complete neurobasal medium was added to neurons.

The neurotoxicity of fibrils was tested after 24 and 48 hours of exposure to fibrils performing MTT (3-(4,5-dimethylthiazol-2-yl)-2,5-diphenyltetrazolium bromide) assay. MTT solution was prepared by dissolving MTT (VWR International) to dulbecco’s phosphate buffered saline (DPBS, Thermo Fisher), pH = 7.4, to a final concentration of 5 mg/mL. To prepare the solubilization solution, 40% v/v dimethylformamide (DMF, Sigma Aldrich) was added to 2% v/v glacial acetic acid (Sigma Aldrich), and 16% w/v sodium dodecyl sulfate (SDS, Hoefer Inc.) was dissolved in the solution, and the pH was adjusted to 4.7. Then, 10 μL of MTT solution was added to untreated and treated neurons with a volume of 200 μL neurobasal medium, and incubated for 3 hours at 37°C. The culture medium then was removed and replaced with solubilization solution to solubilize produced formazan crystals. The cells were incubated with the solubilization solution for 20 minutes, and after incubation, their optical density was measured at 570 nm using Varioskan Lux Microplate Reader (Thermo Fisher Scientific). Optical densities were normalized with the optical density of the control, and reported as the neuron survival (%) in the Fig. 6C. Three replicates of the MTT assay were performed.

**Statistical tests for similarity between groups of data on Figs. 2D, 3D, 4E, 4F, 5D, 6C, and S3.** To test if the growth rates of the normal and bexarotene fibrils in absence and presence of bexarotene at the specified concentrations (Figs. 2D and 5D) are statistically identical, one-way analysis of variance (one-way ANOVA) was employed which compares the variance between each group to the variance within each group.

For normal fibrils, the F-value is 0.32, smaller than critical F-value 2.69 resulted from 4 groups of 34, 29, 27, and 24 individual measurements with a 95% confidence interval. The p-value is 0.81, greater than the α-value of 0.05. The F-value and p-value indicate the null hypothesis is true, which means the mean values are the same for all independent groups. Therefore, the growth rates of normal fibrils are not affected by bexarotene at all indicated concentrations.

We also performed Kruskal-Wallis test for the data in Fig. 2D (Table S5). The obtained p-value of 0.75 is greater than the α-value 0f 0.05 and suggests that the null hypothesis that the mean ranks of the groups are the same is true. This result advocates that bexarotene does not affect the growth rates of normal fibrils, consistent with ANOVA test results.

For bexarotene fibrils, the F-value from two groups of 24 and 36 individual measurements is 0.19, smaller than critical F-value 4.01, and the p-value is 0.66, greater than the α-value of 0.05 (95% confidence interval), suggesting that the two groups are statistically identical. This ratifies that bexarotene does not affect the growth rates of bexarotene fibrils.

The same one-way ANOVA tests were performed for normal and bexarotene fibrils thicknesses measured by AFM, fibril widths, and widths at the crossovers (Table S3). In all cases F-values are greater than critical F-values and p-values are smaller than the α-value of 0.05 (95% confidence interval), rejecting the null hypothesis which suggests the two groups are statistically different.

The two-way ANOVA test was performed for the neurotoxicity measurements. Two categories of normal and bexarotene fibrils have three concentrations of fibrils (10, 20, and 40 μM) each (Table S4). The p-value for normal and bexarotene fibrils categories is greater than α-value of 0.05 for 24-hour treatment and smaller than α-value of 0.05 for 48-hour treatment which means the neurotoxicity of normal and bexarotene fibrils are statistically identical after 24 hours, while, different after 48 hours. The p-values for comparisons between fibrils concentrations are greater than α-value of 0.05 for both 24 and 48-hour treatment, suggesting that increasing fibril concentration does not affect the neurotoxicity of fibrils. Also, the p-values for interaction between two categories are greater than α-value of 0.05 for both 24 and 48-hour treatment, which means null hypothesis cannot be rejected and there is no correlation between neurotoxicity of normal and bexarotene fibrils, and fibril concentrations.

# Supplementary text

**A two-step kinetic model of association of solute peptides to the fibril tips.** Similarly to ref. (10) we model the two-step incorporation of Aβ40 monomers $M$ into fibril tips $T$ with a Michaelis-Menten-type two-reaction sequence,

$$M+T\begin{matrix} k_{1} \\ \rightleftharpoons\\ k_{-1} \end{matrix}MT\begin{matrix} k_{2} \\ \rightleftharpoons\\ k_{-2} \end{matrix}T^{'} ,$$

where $k_{1}$ and $k_{2}$ are the respective rate constants, $MT$ denotes the frustrated complex at the tip, and $T^{'}$ is the tip with an added peptide. Assuming that the concentration of the intermediate complex $MT$ remains steady,

$$0=\frac{d[MT]}{dt}=k_{1}\left[ M \right]\left[ T \right]-k_{-1}\left[ MT \right]-k_{2}\left[ MT \right]+k_{-2}\left[ T' \right].$$

Furthermore, the association of a monomer does not modify the concentration of fibril tips so that $\left[ T^{'} \right]=[T]$. With this, the total tip concentration

$$C_{T}=\left[ T \right]+\left[ MT \right] .$$

Combining the latter two relations and solving for $\left[ MT \right],$ we obtain

$$\left[ MT \right]=\frac{{(k}_{1}\left[ M \right]+k_{-2})C_{T}}{k_{1}\left[ M \right]+k_{-1}+k_{2}+k_{-2}} .$$

On the other hand, the rate of integration of monomers into the fibril tips

$$-\frac{d\left[ M \right]}{dt}=\frac{d\left[ T^{'} \right]}{dt}=k_{2}\left[ MT \right]-k_{-2}\left[ T \right] .$$

Substituting the expression for $\left[ MT \right]$ and summing, we get

$$-\frac{d\left[ M \right]}{dt}=\frac{k_{1}k_{2}\left[ M \right]-k_{-1}k_{-2}}{k_{1}\left[ M \right]+k_{-1}+k_{2}+k_{-2}}C_{T} .$$

This relation is equivalent to Eq. (5b) from ref. (10).

We relate $-d[M]/dt$ to the average rate of growth of fibrils $R$. If a volume $V$ holds $N$ fibril tips, $R_{j}$ is the rate of growth of fibril tip $j$, and $a$ is the contribution of one monomer to the fibril length, then $R_{j}/a$ is the rate of incorporation of monomers into fibril tip $j$. The rates $R_{j}$ tend to be steady in time (Fig. 2C) and then the number of monomers integrated into all tips in the volume $V$ per unit time is

$$\sum_{j}^{N} \frac{R_{j}}{a}=-N_{A}\frac{d\left[ M \right]}{dt}V ,$$

where $N_{A}$ is Avogadro’s number. Summing the left-hand side and using that $\sum R_{j}=NR$ and $C_{T}=N\left( VN_{A} \right)^{-1}$

$$R=-\frac{a}{C_{T}}\frac{d[M]}{dt}$$

and

$$R=a\left( \frac{k_{1}k_{2}\left[ M \right]-k_{-1}k_{-2}}{k_{1}\left[ M \right]+k_{-1}+k_{2}+k_{-2}} \right) .$$

At equilibrium between the tips and the solution, $\left[ M \right]=\left[ M \right]_{e}$, $R$ =0, and $k_{-1}k_{-2}=k_{1}k_{2}\left[ M \right]_{e}$ or

$$\left[ M \right]_{e}=\frac{k_{-1}}{k_{1}}\frac{k_{-2}}{k_{2}}=K_{1}^{-1}K_{2}^{-1}=K^{-1} ,$$

where $K$ is the equilibrium constant for the entire process and $K_{1}$ and $K_{2}$, the equilibrium constants for the two constituent reactions. On the other hand, the equilibrium $T + M \rightleftharpoons T'$ is characterized by a constant $K=\left[ M \right]_{e}^{-1}$ owing to the equality $\left[ T \right]=[T']$ ref. (11). The congruity with the relation between $\left[ M \right]_{e}$ and $K$ resulting from the kinetic model certifies the thermodynamic consistency of the kinetic model.

Finally, considering the dominance of monomers in the solution, we approximate the monomer concentration $[M]$ with the total peptide concentration $C_{A\beta40}$ and $\left[ M \right]_{e}$ with the solubility $C_{e}$ and arrive at

$$R=a\left( \frac{k_{1}k_{2}(C_{A\beta40}-C_{e})}{k_{1}C_{A\beta40}+k_{-1}+k_{2}+k_{-2}} \right) .$$

To determine the parameters that ensure the best fit of the kinetic law to the $R(C_{A\beta40})$ data obtained by AFM we divide both the numerator and denominator by $k_{1}$*,* denote, for brevity, the ratio $\frac{k_{-1}+k_{2}+k_{-2}}{k_{1}}$ as $K_{T}$ and obtain

$$R=a\left( \frac{k_{2}(C_{A\beta40}-C_{e})}{C_{A\beta40}+K_{T}} \right)=\frac{A(C_{A\beta40}-C_{e})}{{(C}_{A\beta40}-C_{e})+B} ,$$

where $A=ak_{2}$ and $B={K_{T}+C}_{e}$. To linearize this relation, we follow the Eadie-Hofstee rearrangement of the Michaelis-Menten law for enzyme kinetics to reach

$$\frac{R}{C_{A\beta40}-C_{e}}= \frac{A}{B}- \frac{R}{B}$$

A plot of $(\frac{R}{C_{A\beta40}-C_{e}})$ as a function of $R$ gives a straight line with slope $-\frac{1}{B}$ and intercept $\frac{A}{B}$. We evaluate the approximate value of the solubility $C_{e}$ by linearly interpolating the $R(C_{A\beta40}$) data for $R$ = 0 (Fig. 5E). The parameters $C_{e}$, $A$, and $B$ for the $R(C_{A\beta40})$ correlations of bexarotene fibrils in the presence and absence of urea are listed in Table S2.

# Supplementary references

1. Walsh, D. M., Thulin, E., Minogue, A. M., Gustavsson, N., Pang, E., Teplow, D. B., and Linse, S. (2009) A facile method for expression and purification of the Alzheimer's disease-associated amyloid beta-peptide. *The FEBS journal* **276**, 1266-1281

2. Strohalm, M., Hassman, M., Košata, B., and Kodíček, M. (2008) mMass data miner: an open source alternative for mass spectrometric data analysis. *Rapid Communications in Mass Spectrometry* **22**, 905-908

3. Zheng, W., Tsai, M.-Y., Chen, M., and Wolynes, P. G. (2016) Exploring the aggregation free energy landscape of the amyloid-β protein (1–40). *Proceedings of the National Academy of Sciences* **113**, 11835-11840

4. Meisl, G., Kirkegaard, J. B., Arosio, P., Michaels, T. C. T., Vendruscolo, M., Dobson, C. M., Linse, S., and Knowles, T. P. J. (2016) Molecular mechanisms of protein aggregation from global fitting of kinetic models. *Nature Protocols* **11**, 252-272

5. Shen, C. L., and Murphy, R. M. (1995) Solvent effects on self-assembly of beta-amyloid peptide. *Biophysical journal* **69**, 640-651

6. Batista, A. N. L., Batista Jr, J. M., Bolzani, V. S., Furlan, M., and Blanch, E. W. (2013) Selective DMSO-induced conformational changes in proteins from Raman optical activity. *Physical Chemistry Chemical Physics* **15**, 20147-20152

7. Habchi, J., Arosio, P., Perni, M., Costa, A. R., Yagi-Utsumi, M., Joshi, P., Chia, S., Cohen, S. I., Müller, M. B., Linse, S., Nollen, E. A., Dobson, C. M., Knowles, T. P., and Vendruscolo, M. (2016) An anticancer drug suppresses the primary nucleation reaction that initiates the production of the toxic Aβ42 aggregates linked with Alzheimer's disease. *Sci Adv* **2**

8. Xu, Y., Safari, M. S., Ma, W., Schafer, N. P., Wolynes, P. G., and Vekilov, P. G. (2019) Steady, Symmetric, and Reversible Growth and Dissolution of Individual Amyloid-β Fibrils. *ACS Chemical Neuroscience* **10**, 2967-2976

9. Sherman, M. B., Guenther, R., Reade, R., Rochon, D. A., Sit, T., Smith, T. J., and Parrish, C. R. (2020) Near-Atomic-Resolution Cryo-Electron Microscopy Structures of Cucumber Leaf Spot Virus and Red Clover Necrotic Mosaic Virus: Evolutionary Divergence at the Icosahedral Three-Fold Axes. *Journal of Virology* **94**, e01439-01419

10. Qiang, W., Kelley, K., and Tycko, R. (2013) Polymorph-Specific Kinetics and Thermodynamics of β-Amyloid Fibril Growth. *Journal of the American Chemical Society* **135**, 6860-6871

11. Xu, Y., Knapp, K., Le, K. N., Schafer, N. P., Safari, M. S., Davtyan, A., Wolynes, P. G., and Vekilov, P. G. (2021) Frustrated peptide chains at the fibril tip control the kinetics of growth of amyloid-β fibrils. *Proceedings of the National Academy of Sciences* **118**, e2110995118

# Supplementary Figures


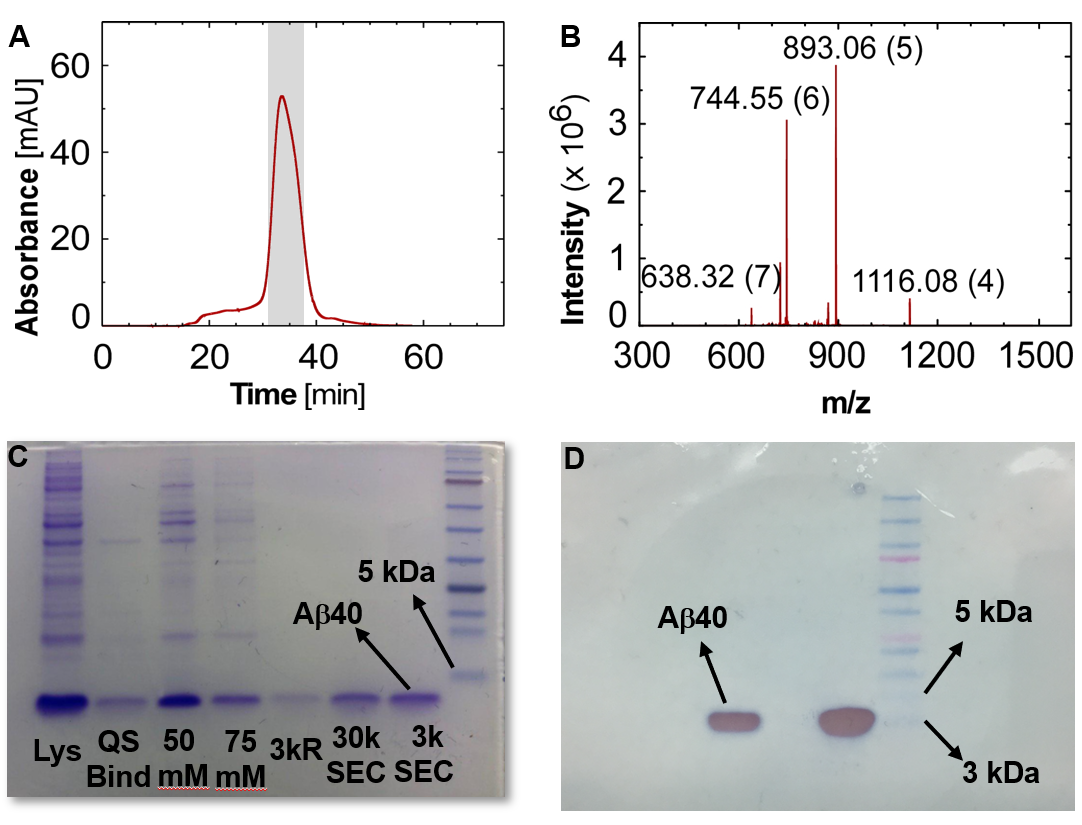


**Fig. S1. Purification and identification of Aβ40 peptide.** (**A**) Size exclusion chromatogram (SEC) of Aβ40 separated from 3 kDa MWCO retentate; the peak at 32 minutes that corresponds to the Aβ40 peptide. The UV absorbance at 280 nm was measured by the built-in detector of ÄKTA Pure System. Grey band highlights fraction used in fibrillization experiments. (**B**) The mass spectrum of the Aβ40 peptide. The major peaks with their m/z ratio are displayed. Numbers in parenthesis indicate the charge carried by the molecules. (**C**) Electrophoresis on a 4%-12% Bis-Tris gel of fractions collected from different purification steps. Rightmost lane displays protein molecular weight standards (Position Plus Protein Dual Xtra, BIO-RAD). Lowest band in each lane corresponds to Aβ40. Lanes from left to right: cell lysate after solubilization with urea (Lys), supernatant after binding cell lysate to Q-sepharose (QS Bind), elutions using buffers with 50 and 75 mM NaCl, retentate of 3 kDa MWCO filtration (3kR), SEC peak fraction of Aβ40 peptide separated from 30 kDa MWCO retentate (30k SEC), and SEC peak fraction of Aβ40 peptide separated from 3 kDa MWCO retentate (3k SEC). (**D**) Western blot results. Right lane displays the protein molecular weight standards, left lane the SEC fraction same as 3kR in C, and middle lane the concentrated fraction of the SEC fraction. The appearance of colored fragments in both lanes confirms the presence of Aβ40 peptide.

**
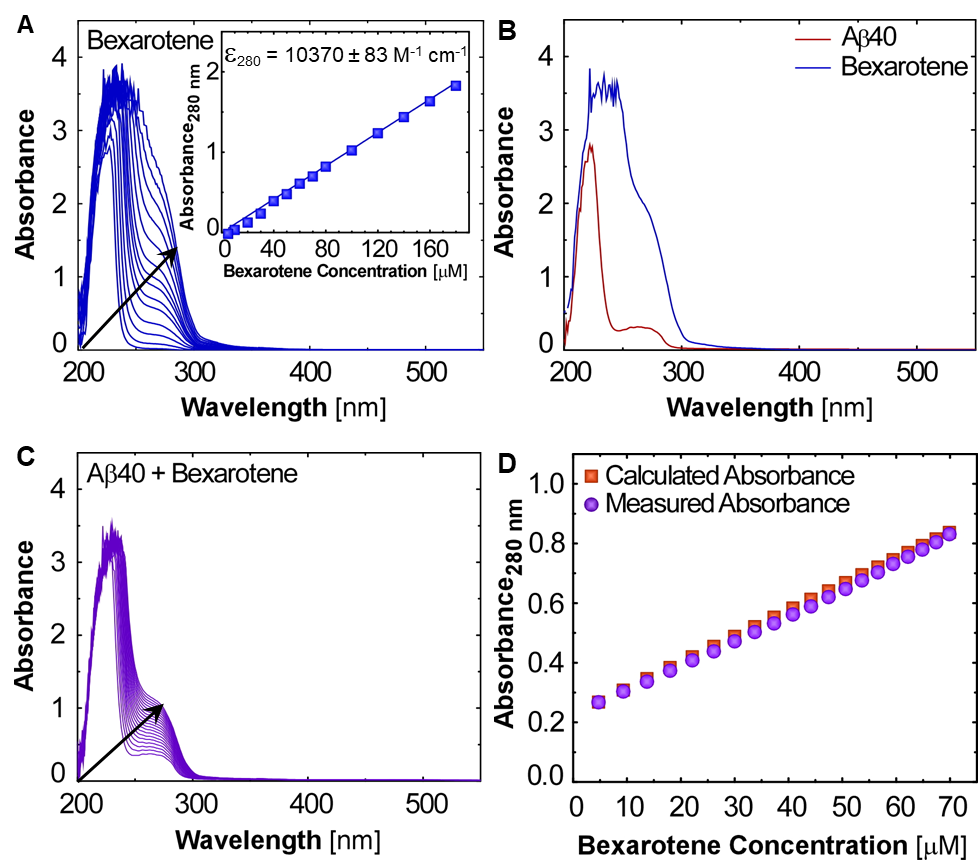
**

**Fig. S2. Lack of complexation of Aβ40 peptides with bexarotene.** (**A**) UV-Vis spectra of bexarotene in ammonium acetate buffer at several concentrations between 5 and 180 μM. The inset shows the correlation between UV absorbance at 280 nm and bexarotene concentration. The slope of the best linear regression fit determines the reported extinction coefficient ε_280_. Arrow indicates direction of increasing bexarotene concentration. (**B**) The spectra of bexarotene at 140 μM (blue) and Aβ40 peptide at 156 μM (red). (**C**) Variation of the UV-Vis spectrum of Aβ40 upon the addition of bexarotene. The initial concentration of Aβ40 is 156 μM and bexarotene is added in 20 aliquots of 20 μl. The final concentration of bexarotene in the solution is 70 μM. Arrow indicates direction of increasing bexarotene concentration. (**D**) The absorbance of the Aβ40-bexarotene solution measured at 280 nm (purple spheres) as a function of bexarotene concentration compared with calculated sum of Aβ40 and bexarotene absorbance (orange squares) at each bexarotene concentration, assuming no complex is formed. The similarity of the measured and calculated absorbances advocates lack of complexation of Aβ40 peptides with bexarotene.

**
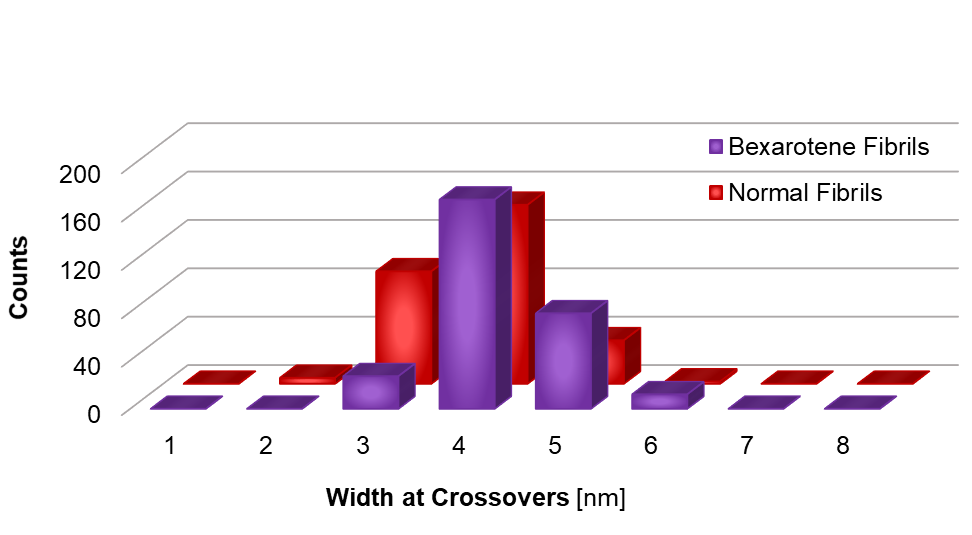
**

**Fig. S3.** Distribution of the widths at the crossover points for normal (red) and bexarotene (purple) fibrils.


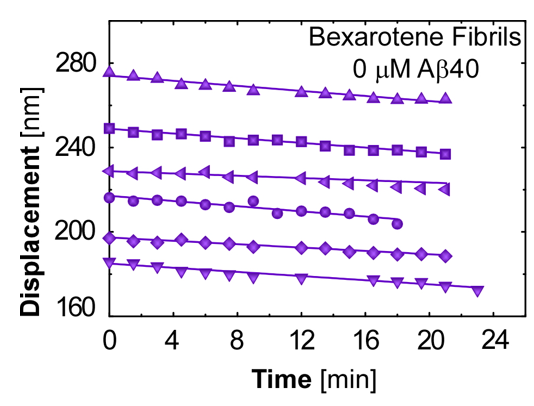


**Fig. S4. Dissolution of bexarotene fibrils.** The displacements of six bexarotene fibril tips dissolving in peptide-free solutions. The slopes of the best-fit lines determine the average fibril dissolution rates.

**
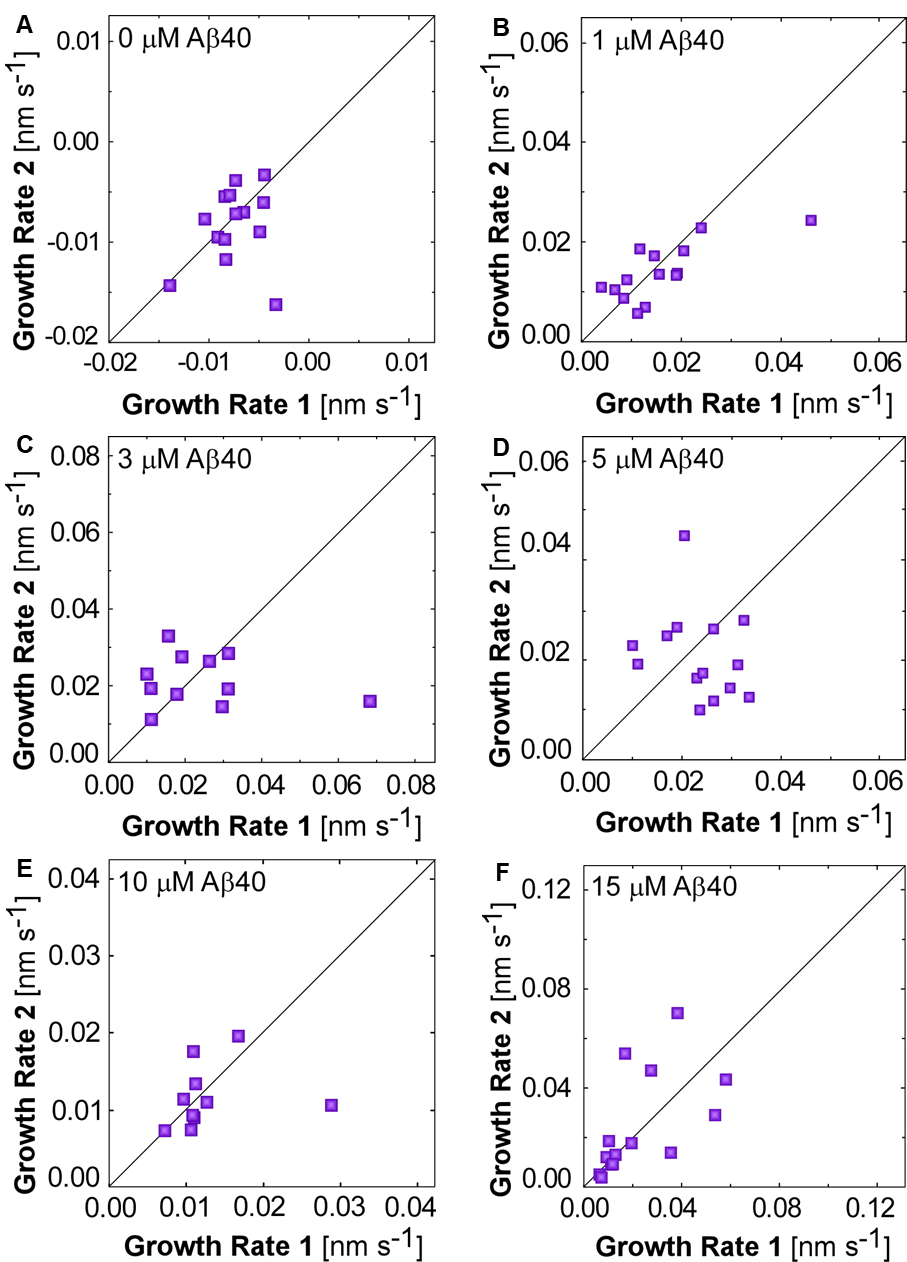
**

**Fig. S5. Asymmetric growth of bexarotene fibril ends.** (**A-F**) Correlations between growth rates of the two ends of bexarotene fibrils at indicated Aβ40 concentrations.

**
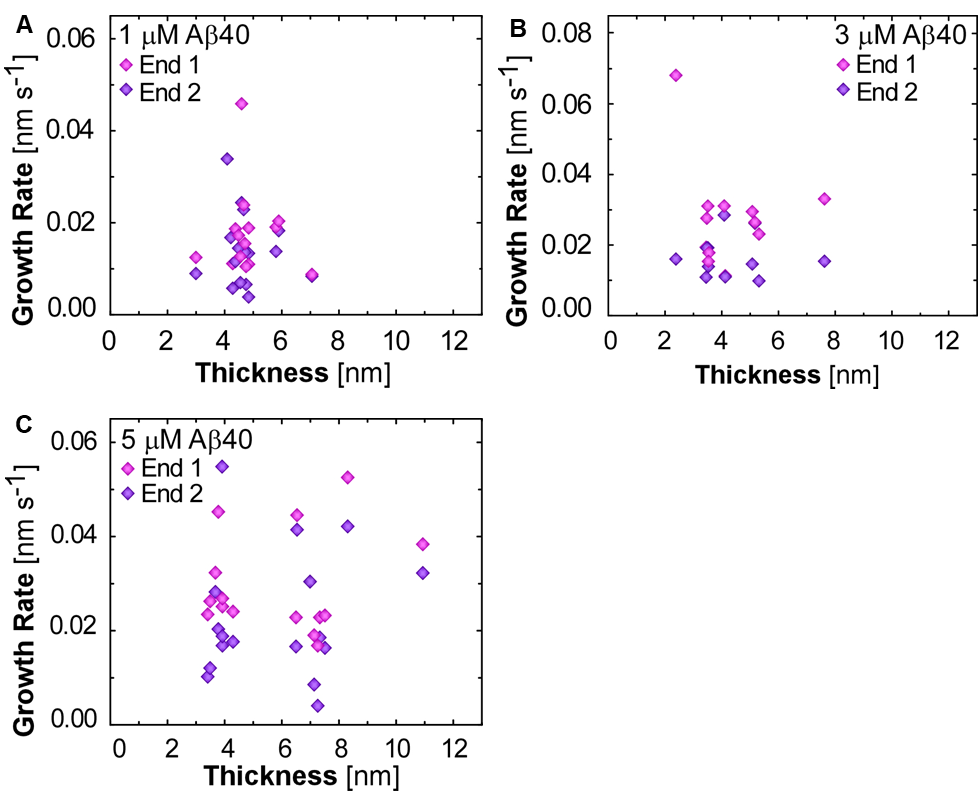
**

**Fig. S6. Lack of correlation between growth rates bexarotene fibrils and the fibrils thickness.** (**A-C**) Growth rates of the opposing bexarotene fibril ends as a function of the respective fibril thickness at indicated Aβ40 concentrations.

# Supplementary Tables

**Table S1. Summary of the structural characteristics of normal and bexarotene fibrils**

| **Fibril Type** | | **Crossover Distance [nm]** | **Fibril Width  [nm]** | **Width at Crossovers [nm]** |
| --- | --- | --- | --- | --- |
| **Normal Fibrils** | | 142 ± 21 | 8 ± 1 | 4 ± 1 |
| **Bexarotene**  **Fibrils** | **Short Crossovers** | 25 ± 4 | 10 ± 2 | 5 ± 1 |
|  | **Long Crossovers** | 134 ± 33 |  |  |

**Table S2. The parameters that ensure the best fit of the kinetic model to the** $\boldsymbol{R(}\boldsymbol{C}_{\boldsymbol{A\beta}\boldsymbol{40}}\boldsymbol{)}$ **correlations for bexarotene fibrils**

| **Bexarotene**  **Fibrils** | **C_e_ [μM]** | **Slope** | **Intercept** | **A [nm s^-1^]** | **B [μM]** | **k_2_ [s^-1^]** | **K_T_ [μM]** |
| --- | --- | --- | --- | --- | --- | --- | --- |
| 0 M Urea | 0.33 ±0.06 | 0.54 ±0.32 | 0.02 ±0.01 | 0.04 ±0.02 | 1.85 ±0.90 | 0.08 ±0.02 | 1.52 ±0.90 |
| 1 M Urea | 0.45 ±0.07 | 0.62 ±0.26 | 0.03 ±0.01 | 0.05 ±0.02 | 1.61 ±0.49 | 0.1 ±0.02 | 1.16 ±0.48 |

**Table S3. The analysis of variance (ANOVA) test parameters**

| **Source of Variation** | **Sum of Squares** | **Degrees of Freedom** | **Mean Squares** | **F** | **p-value** | **F critical** |
| --- | --- | --- | --- | --- | --- | --- |
| **Effect of bexarotene on normal fibrils growth rates (Fig. 2D)** | | | | | | |
| Between Groups | 0.0001 | 3 | 5E-05 | 0.32 | 0.81 | 2.69 |
| Within Groups | 0.0166 | 110 | 0.0002 |  |  |  |
| Total | 0.0167 | 113 | Confidence Interval 95% | | | |
| **Effect of bexarotene on bexarotene fibrils growth rates (Fig. 5D)** | | | | | | |
| Between Groups | 3.45E-05 | 1 | 3.45E-05 | 0.19 | 0.66 | 4.01 |
| Within Groups | 0.0103 | 58 | 1.78E-04 |  |  |  |
| Total | 0.0103 | 59 | Confidence Interval 95% | | | |
| **AFM-determined thicknesses of normal and bexarotene fibrils (Fig. 3D)** | | | | | | |
| Between Groups | 70.43 | 1 | 70.43 | 31.74 | 1.09E-07 | 3.92 |
| Within Groups | 279.58 | 126 | 2.22 |  |  |  |
| Total | 350.01 | 127 | Confidence Interval 95% | | | |
| **Widths at crossovers of normal and bexarotene fibrils (Fig. S3)** | | | | | | |
| Between Groups | 24.61 | 1 | 24.61 | 58.19 | 9.97E-14 | 3.86 |
| Within Groups | 242.36 | 573 | 0.42 |  |  |  |
| Total | 266.98 | 574 | Confidence Interval 95% | | | |
| **Fibril widths of normal and bexarotene fibrils (Fig. 4F)** | | | | | | |
| Between Groups | 340.68 | 1 | 340.68 | 144.45 | 2.19E-29 | 3.86 |
| Within Groups | 1172.20 | 497 | 2.36 |  |  |  |
| Total | 1512.88 | 498 | Confidence Interval 95% | | | |
| **Neurotoxicity of normal and bexarotene supernatants (Fig. 6C, 24 hours)** | | | | | | |
| Between Groups | 9.46 | 1 | 9.46 | 0.79 | 0.47 | 18.51 |
| Within Groups | 23.92 | 2 | 11.96 |  |  |  |
| Total | 33.38 | 3 | Confidence Interval 95% | | | |
| **Neurotoxicity of normal and bexarotene supernatants (Fig. 6C, 48 hours)** | | | | | | |
| Between Groups | 0.04 | 1 | 0.04 | 0.28 | 0.65 | 18.51 |
| Within Groups | 0.28 | 2 | 0.14 |  |  |  |
| Total | 0.32 | 3 | Confidence Interval 95% | | | |

**Table S4. The two-way ANOVA tests of the similarities of the neuron survival measurements (Fig. 6C)**

| **Source of Variation** | **Sum of Squares** | **Degrees of Freedom** | **Mean Squares** | **F** | **p-value** | **F critical** |
| --- | --- | --- | --- | --- | --- | --- |
| **24 hours** | | | | | | |
| Bexarotene/Normal | 938.72 | 1 | 938.72 | 3.69 | 0.10 | 5.99 |
| Fibril Concentration | 456.22 | 2 | 228.11 | 0.90 | 0.46 | 5.14 |
| Interaction | 106.97 | 2 | 53.49 | 0.21 | 0.82 | 5.14 |
| Total | 3029.51 | 11 |  |  |  |  |
| **48 hours** | | | | | | |
| Bexarotene/Normal | 395.73 | 1 | 395.73 | 10.75 | 0.02 | 5.99 |
| Fibril Concentration | 105.32 | 2 | 52.66 | 1.43 | 0.31 | 5.14 |
| Interaction | 127.51 | 2 | 63.75 | 1.73 | 0.25 | 5.14 |
| Total | 849.42 | 11 |  |  |  |  |

**Table S5. The parameters of the Kruskal-Wallis test of the effect of bexarotene on normal fibrils growth rates (Fig. 2D).**

| **Source of Variation** | **n** | **Mean Rank** | **Degrees of Freedom** | **H** | **p-value** | **χ^2^ critical** |
| --- | --- | --- | --- | --- | --- | --- |
| 0 μM Bexarotene | 34 | 57.91 | 3 | 1.20 | 0.75  (α = 0.05) | 7.81 |
| 0.1 μM Bexarotene | 29 | 62.66 |  |  |  |  |
| 0.5 μM Bexarotene | 27 | 54.61 |  |  |  |  |
| 1 μM Bexarotene | 24 | 53.94 |  |  |  |  |
